# Supplementary material for: Rapid antigen testing by community health workers for detection of SARS-CoV-2 in Dhaka, Bangladesh: a cross-sectional study
Source: BMJ Open. 2022 Jun 1;12(6):e060832. doi: 10.1136/bmjopen-2022-060832 (PMC9160589; doi:10.1136/bmjopen-2022-060832)
Supplement: Supplementary data [file bmjopen-2022-060832supp001.pdf]

Overall procedure

1. CSTs will follow their normal routine aiming to find potential virus fighters (PVFs). Once the PVF is found, the CST will perform PVF screening (follow SOP 03) and, if appropriate, VVF screening (follow SOP 05) for verified virus fighter (VVF) identification.
2. If the VVF is found, the team should provide regular support. The VVF and 2 additional household (HH) members (if present) should be informed about the study and the written consent must be obtained before any sample collection.
3. During sample collection and rapid antigen testing the CST team will share responsibilities.
   - CST1 will be collecting rapid test samples (step1) and sample for PCR test (step2) as well as the metadata using the IEDCR app
   - CST2 will perform rapid antigen tests on samples collected by CST1. CST2 will also enter corresponding data into the electronic Kobo forms
4. First, CST1 will collect saliva samples and nasal swabs from all 3 household members (6 samples in total)
5. CST1 will pass the samples (labelled with barcodes and indicating sample type) to CST2 who will perform rapid antigen tests (follow instructions below)
6. While the rapid tests run, CST1 will collect 3 additional samples for PCR (for the VVF and each of the additional HH members) and collect all necessary information (metadata).
7. When the results of rapid test are visible - CST2 will fill out the Kobo form
8. If the rapid test is positive for any of the HH members the household should be instructed to self-isolate . If the rapid test is negative for all HH members the household should also be instructed to quarantine until PCR results are sent (by SMS) and only if the negative result is confirmed can they stop quarantine after 14 days from last exposure .

TIMELINE: after VVF is identified sample collection will follow the workflow:

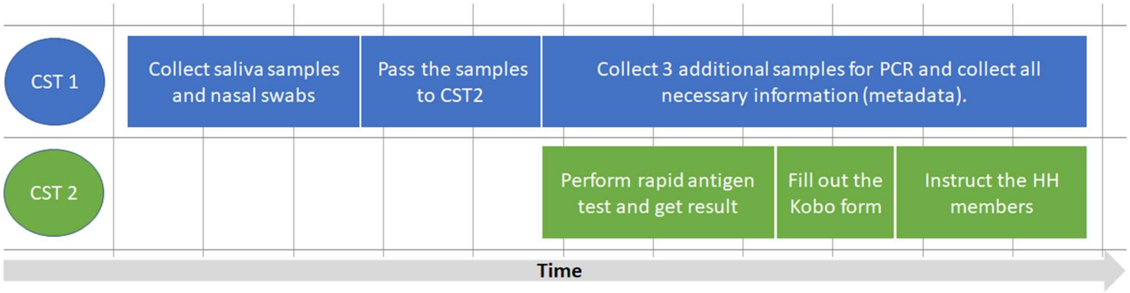

Pre-Requisites

Fifteen (15) MTs will be hired and trained for this phase of the study. Their primary role will be assisting and mentoring CSTs in their activities. After one month of working together the CSTs should gain sufficient confidence to collect samples independently.

IEDCR's Medical Technologist (MT) will join a CST team (two CST members). CST will follow their daily routine finding PVFs and identifying VVFs by following their SOPs. Once the VVF is identified rapid antigen tests will be performed by the CSTs (on saliva sample and on nasal swab). Additionally, CSTs will collect PCR samples to be tested at the IEDCR and all necessary metadata.

The MT MUST NOT interfere with CST activities. MT should ensure all samples are collected properly. MT should be a mentor for the CSTs, whose aim is to become independent after one month's time period. MT can advise the CST on rapid antigen results interpretation but MUST NOT perform the test.

Considering that the above prerequisites are met, the rest of this document describes the step-by-step procedures for the CSTs and MTs to collect, record, store, transport, and test the samples as well as report the results. In this document, subjects requesting a test will be referred to as ***patients,*** while the patients testing positive with RT-PCR for COVID-19 will be referred to as ***cases***.

Preparation

Preparation

Before leaving for sample collection, the MTs/CSTs will collect the following from the IEDCR office.

☐	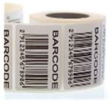	Eight (8) printed copies of seven (7) digit barcode labels for each allocated patient (1 for labeling the Viral transporting medias (VTMs) for PCR testing, 3 for the saliva collection tube, extraction buffer tube (salive to be added into) and the corresponding saliva rapid test, 2 for the nasal swab extraction buffer tube and the corresponding rapid antigen test, 2 for the informed consent forms)
☐	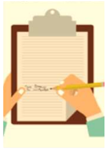	Informed consent forms to get the patient’s consent. Put a barcode label on the top right corner of the form.
☐	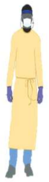	Sealed PPEs (1 for attending each patient)

☐	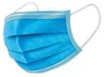	Disposable masks
☐	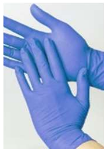	Disposable gloves
☐	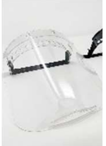	Face shield
☐	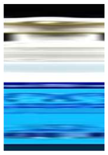	Disinfectant
☐	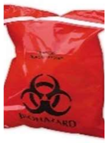	Biohazard bag for disposal of used PPEs, gloves, masks
☐	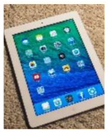	A digital tab for inserting the patients' information into the app
☐	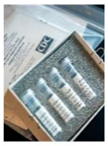	VTMS
☐	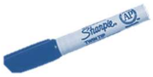	marker for labeling VTMs, tubes and rapid tests

☐	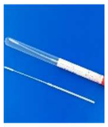	Nasal and throat swab collectionkits
☐	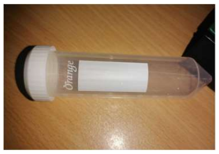	Saliva collection kits (screw cap container)

During the household visit the CST team will follow their routine and perform the following activities:

- PVF screening
- VVF identification

If the PVF (one per HH) is identified as VVF the CST1 will collect their saliva and nasal swab samples and perform rapid antigen tests. In addition, the CST will collect PCR samples (O+N swabs). Rapid antigen and PCR samples will be collected from additional two members of the household who are present during the visit (giving priority to those with any covid-like symptoms and/or to those with being the closest contacts of the VVF).

Explaining the study and collecting the consent

☐	CST will inform that this is a routine test from IEDCR to identify covid-19 cases. In addition to the routine nasal and throat samples (PCR), two more samples will be collected: nasal sample and saliva sample
☐	Notify that the participation is voluntary. If a patient refuses to participate/give additional samples, the routine sample collection procedure will be carried out and all information will be collected in the **IEDCR case selection app** in the digital tab.
☐	Notify that the collection of biospecimens does not pose any potential safety risk.
☐	Notify that these tests do not usually hurt but may cause a little discomfort.
☐	Notify that the maintenance of data confidentiality will be strictly followed, and restrictions on access to data forms will be ensured.

Notify that the patient will be informed about the PCR result by text message or email.

☐ If the rapid test is positive for any of the HH members the household should be instructed to quarantine. If the rapid test is negative, the household should be instructed to self-isolate until PCR results are sent (by SMS) and only if all are negative can they stop isolation.

Labelling sample collection tubes and tests

After obtaining the consent, CST2 will label

- 1,2 or 3 (depending on number of HH members to be tested) rapid antigen tube/s (extraction buffer tube/s) with different barcodes.And also label the tube/s with “+S” (which refers to saliva sample).
- Appropriate number of rapid antigen tubes (extraction buffer tubes) with the corresponding barcodes. And also label the tube/s with “+N” (which refers to nasal sample).
- Same number of falcon tubes (large ones for saliva collection) with the corresponding barcodes
- Same number of VTMs (for PCR sample collection) with the corresponding barcodes
- The sample collection tubes with the VVF’s and HH members’ names to prevent any confusion.
- Using corresponding barcodes rapid antigen tests will be labelled as well (adding S for saliva and N for nasal swab sample)

PPE

CST1 and CST2 will now

☐ **disinfect hands**

CST1 will wear the PPE from the sealed bag, disposable mask, and face shield.

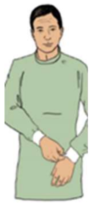	☐ PPE gown
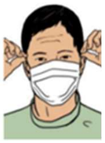	☐ Surgical/N95 mask
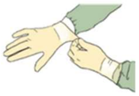	☐ Disposable gloves
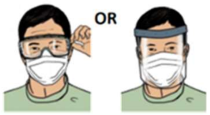	☐ Face shield or goggles

Source: https://www.dph.illinois.gov/sites/default/files/COVID19/COVID19%20sample%20collection%20procedures_5.31.20.pdf
https://www.who.int/csr/disease/plague/collecting-sputum-samples.PDF

Raw Saliva Sample Collection Protocol for Phase 2 (to be collected by CST1)

CST1 should confirm that the patient had no food or drinks before sample collection. Ideally water should be avoided for 10 mins prior to collection and drinks/food/nasal sprays for half an hour before collection. While the patient is getting prepared for the process, CSTs will prepare as well (see above). That way if the patient already had a meal, it will create a time difference for sample collection.

Raw saliva sample collection instruction for CST

The CST2 will hand in the falcon tube with pateind code (RAT 0XXX S) to CST1 for sample collection

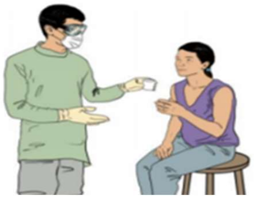

☐ Instruct the patient to think about saliva inducing food such as tamarind and begin pooling saliva in their mouth, the patient can gently massage their cheeks to help the process.

☐ Request the patient not to cough and produce sputum in the mouth.

☐ Labelled (RAT0XXX S) sterile, leak-proof screw cap containers will be handled to the patient.

☐ Patient will be instructed to not to touch the inside of the container.

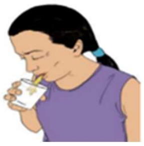

☐ Patient will be instructed to gently expel saliva into the container.

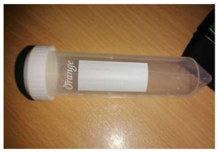

☐ Collect 1-5 mL of saliva.

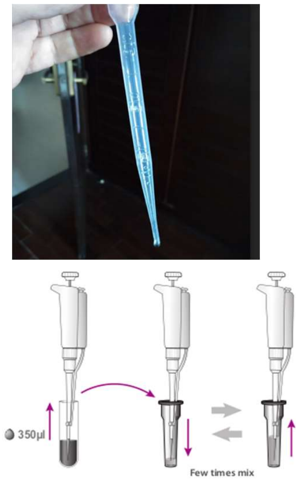

☐ Using an eyedropper collect ~350µl of saliva from the collection tube into the extraction buffer tube (kit) . Mix with an extraction buffer.

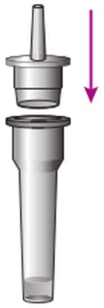

☐ Press the nozzle cap tightly onto the tube

☐ Hand the sample to CST2 to perform the test

Nasal Swab Sample Collection Protocol for Phase 2 (to be collected by CST1)

Nasal sample collection (infographics showing Left Nasal sample collection) instruction for CST

The CST2 will hand in the extraction buffer tube with patient code (RAT 0XXX N) to CST1 for sample collection

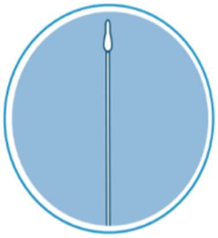

- ☐ Take out the nasal mid-turbinate swab from the packet and keep the tube safely for the time being.
- ☐ Touch only the plastic shaft not the padded end.

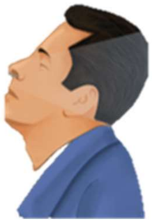

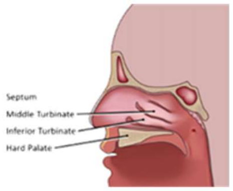

- Ask the patient to sit straight and tilt the head back (approximately 70 degree).

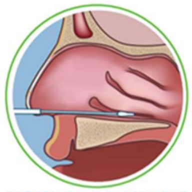

PROPER SWAB PLACEMENT

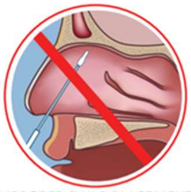

IMPROPER SWAB PLACEMENT

- Insert the swab in the RIGHT nasal spare parallel to the hard palate.
- Resistance will be felt and that is the confirmation of reaching to the nasopharynx.

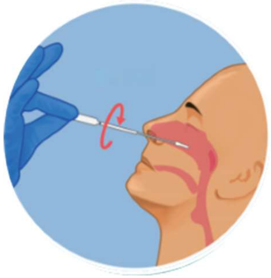

- Once the swab is against the hard surface rotate it several times.

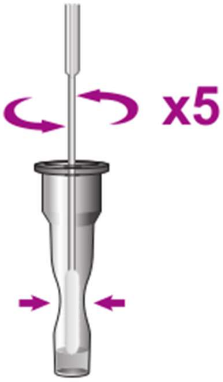

- ☐ Make sure the tube is labelled correctly (RATXXX N, where the barcode corresponds to the right patient)
- ☐ Insert the swab into an extraction buffer tube. While squeezing the buffer tube, stir the swab more than 5 times.
- ☐ Remove the swab while squeezing the sides of the tube to extract the liquid from the swab.

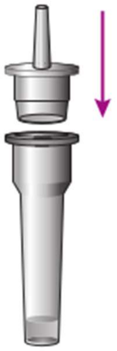

- ☐ Press the nozzle cap tightly onto the tube.
- ☐ Sample to be handed to the CST2

Image and Information Sources:

https://www.cdc.gov/coronavirus/2019-ncov/downloads/lab/NMT_Specimen_Collection_Infographic_FINAL_508.pdf

Rapid antigen test and interpretation of the results (to be performed by CST2)

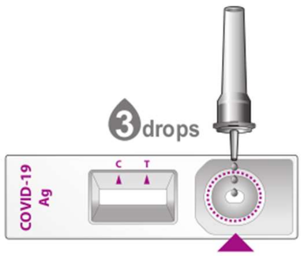

- CST2 will apply 3 drops of extracted specimen to the specimen well of the test device
- double check that the barcode on the tube and n the test is the same (!)

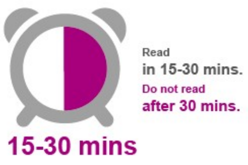

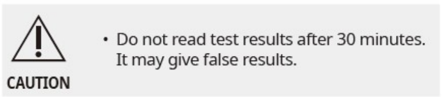

- Read the test result in 15-30 minutes.

Results Interpretation

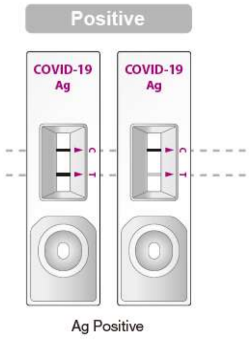

- Presence of a second colored band, "T" test line, in conjunction with the "C" Control line is always considered as positive. **Even if the "T" test line is faint.**
- A colored band, control line (C), in the top section of the result window will appear in positive and negative test results.

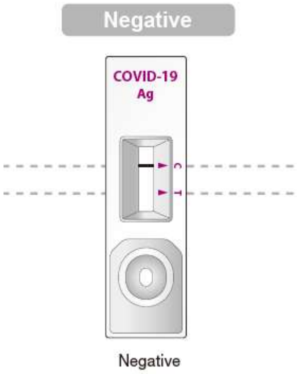

- Presence of only the "C" control line without the "T" test line will be considered as negative.

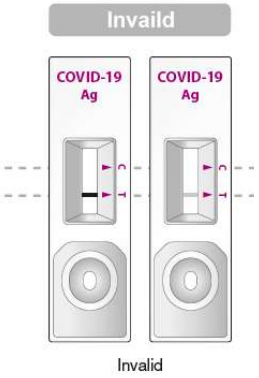

- Absence of the control line in the top section will always consider the result as invalid.

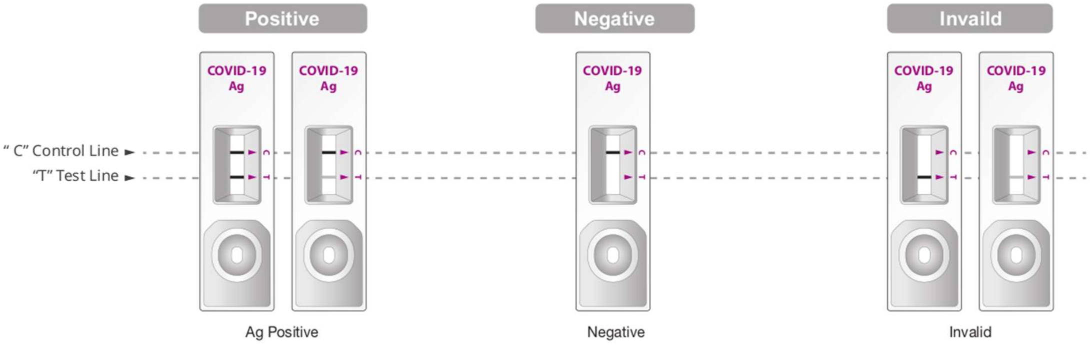

☐ Once both tests are performed and the results are read, CST2 will use Kobo form to register the results, which will involve taking pictures of the tests with visible barcodes for the record.

PCR Sample Collection Protocol for Phase 2 (to be collected by CST1)

PCR sample contains two swabs (OP/throat and nasal), collected into one VTM tube and transported to the IEDCR for PCR test.

Demographic data

Before sample collection, the CST1 will start collecting demographic information in the digital tablet. It is recommended that CST ensure social distance from the patients and keep wearing PPE the entire time during the data collection.

Table: Procedure for inserting information in the digital tablet is as follows:

- ☐ Open the ***RAT study***, ***IEDCR Case selection*** app in the tab.
- ☐ Start a new form.
- ☐ Enter the barcode.
- ☐ Collect and enter patient-specific information in the form. The form contains fields related to patient demography, list of symptoms, date of onset, and any other relevant information. See the screen shots of the form below.
- ☐ Make sure that all available information is collected from the patient and inserted into the form.

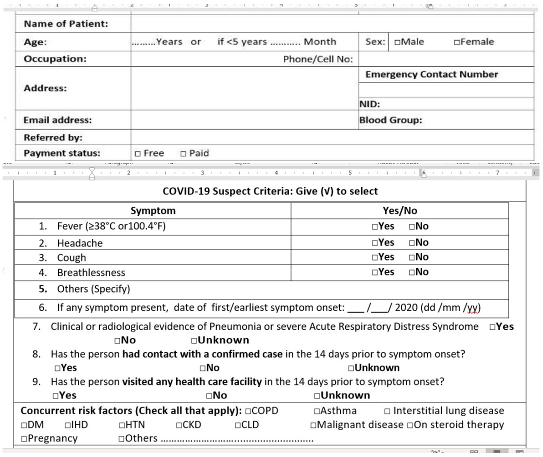

**Name of Patient:**			
**Age:**	………Years or if <5 years ……….. Month	Sex:	□Male □Female
**Occupation:**	Phone/Cell No:		
**Address:**		**Emergency Contact Number**	
		**NID:**	
**Email address:**		**Blood Group:**	
**Referred by:**			
**Payment status:**	□ Free □ Paid		

COVID-19 Suspect Criteria: Give (√) to select

Symptom	Yes/No
1. Fever (≥38°C or100.4°F)	□**Yes** □**No**
2. Headache	□**Yes** □**No**
3. Cough	□**Yes** □**No**
4. Breathlessness	□**Yes** □**No**
**5.** Others (Specify)	
6. If any symptom present, date of first/earliest symptom onset: ___ /___/ 2020 (dd /mm /yy)	

7. Clinical or radiological evidence of Pneumonia or severe Acute Respiratory Distress Syndrome □**Yes** □**No** □**Unknown**
8. Has the person **had contact with a confirmed case** in the 14 days prior to symptom onset? □**Yes** □**No** □**Unknown**
9. Has the person **visited any health care facility** in the 14 days prior to symptom onset? □**Yes** □**No** □**Unknown**

Concurrent risk factors (Check all that apply): □COPD □Asthma □ Interstitial lung disease □DM □IHD □HTN □CKD □CLD □Malignant disease □On steroid therapy □Pregnancy □Others ……………………………………………………

STEP1: OP / Throat swab collection instruction for CST1 (see the infographic below)

The CST2 will hand in the VTM with pateind code (RAT 0XXX) to CST1 for sample collection

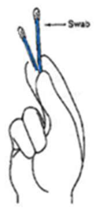

- ☐ Take out one swab from the packet and keep the tubes safely for the time being.
- ☐ Touch only the plastic shafts not the padded ends.

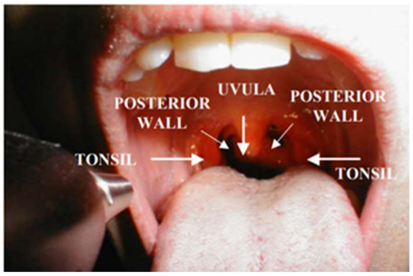

- ☐ Ask the patient to sit straight and tilt the head back.
- ☐ Instruct the patient to widely open the mouth and say “Ahh…”
- ☐ If needed use a sterile tongue depressor to get a better view of the throat.

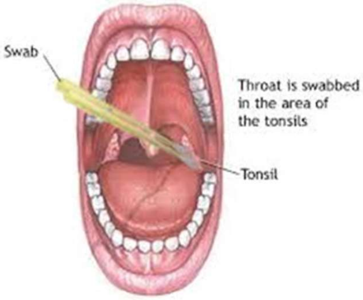

- ☐ Without touching the teeth, gum, tongue, or palate, insert the swabs together to the tonsil and posterior pharynx of any one side.
- ☐ Rub the swabs in the tonsillar area and posterior pharynx, rotate it for 2 times.

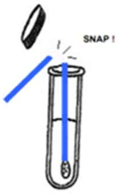

- ☐ After collecting the sample insert the swab into a separate VTMs, label with patient’s barcode
- ☐ Make sure the liquid transport medium covers the tip of the swabs.
- ☐ Break the swab shafts at the marking on the shaft.

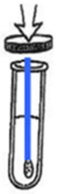

- ☐ Screw the cap back on the test tube tightly.
- ☐ Keep the tube, waiting for inserting a nasal sample for the RT-PCR testing.

STEP2: Nasal sample collection (infographics showing Left Nasal sample collection) instruction for CST1

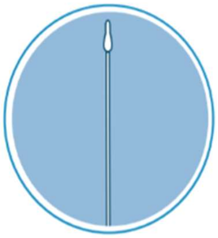

☐ Take out the nasal mid-turbinate swab from the packet and keep the tube safely for the time being.

☐ Touch only the plastic shaft not the padded end.

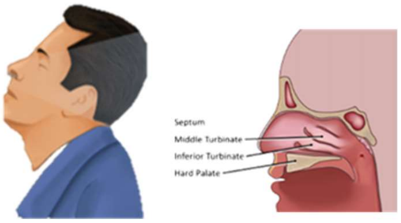

☐ Ask the patient to sit straight and tilt the head back (approximately 70 degree).

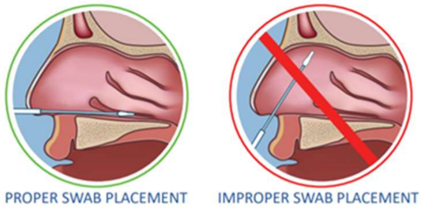

☐ Insert the swab in the LEFT nasal spare parallel to the hard palate.

☐ Resistance will be felt and that is the confirmation of reaching to the nasopharynx.

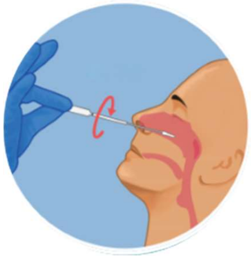

☐ Once the swab is against the hard surface rotate it several times.

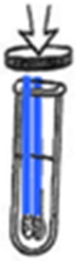

☐ Take out the swab from the left nose and insert the swab into the VTM labelled as "T+N" which already contains the throat swab.

☐ Make sure the liquid transport medium covers the tip of the swabs.

☐ Break the swab shafts at the marking on the shaft.

☐ Screw the cap back on the test tube tightly.

☐ Keep the test tube in the storage box. This combined sample will go for RT-PCR testing

☐ Once the sample is collected, CST1 will check the box in the app (See example below).

Specimen:

☐ Collected ☐ Not collected

If collected mention type:

☐ Combined left nasal swab and throat swab ☐ Nasal swab ☐ Saliva

Image and Information Sources: https://www.cdc.gov/coronavirus/2019-ncov/downloads/lab/NMT_Specimen_Collection_Infographic_FINAL_508.pdf

Sample Store and Transport

- All test tubes/vials to be stored in a Cold Box at 2-8C.
- The samples to be returned to the IEDCR lab at the end of the day.
- Upon return, the storage box will be handled by the lab technicians who run the PCR tests.

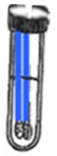

☐ Only the VTM with double swabs, labelled as "T+N",will go for the RT-PCR testing.

At the end of sample collection

CSTs and MT should do the following:

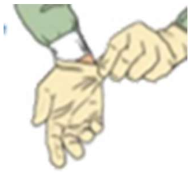	☐ Discard the gloves.
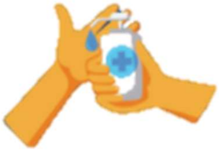	☐ Disinfect the hands with the sanitizer.
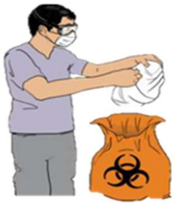	☐ Before entering the transport, CST and MT will discard disposable masks, gloves, PPE in the biodegradable bag. Also disinfect hands as well as the face shield which will be used for the next visit.
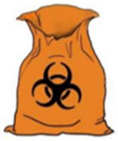	☐ CST and MT must remember that the biodegradable bag needs to be destroyed in the designated place at the IEDCR office.

Image and Information Sources: https://www.who.int/csr/disease/plague/collecting-sputum-samples.PDF
