# Supplementary material for: Rapid antigen testing by community health workers for detection of SARS-CoV-2 in Dhaka, Bangladesh: a cross-sectional study
Source: BMJ Open. 2022 Jun 1;12(6):e060832. doi: 10.1136/bmjopen-2022-060832 (PMC9160589; doi:10.1136/bmjopen-2022-060832)
Supplement: Author's manuscript [file bmjopen-2022-060832.draft_revisions.pdf]

BMJ Open

BMJ Open is committed to open peer review. As part of this commitment we make the peer review history of every article we publish publicly available.

When an article is published we post the peer reviewers' comments and the authors' responses online. We also post the versions of the paper that were used during peer review. These are the versions that the peer review comments apply to.

The versions of the paper that follow are the versions that were submitted during the peer review process. They are not the versions of record or the final published versions. They should not be cited or distributed as the published version of this manuscript.

BMJ Open is an open access journal and the full, final, typeset and author-corrected version of record of the manuscript is available on our site with no access controls, subscription charges or pay-per-view fees (http://bmjopen.bmj.com).

If you have any questions on BMJ Open's open peer review process please email info.bmjopen@bmj.com

BMJ Open

Rapid Antigen Testing by Community Health Workers to improve detection of SARS-CoV-2 in Dhaka, Bangladesh

Journal:	*BMJ Open*
Manuscript ID	bmjopen-2022-060832
Article Type:	Original research
Date Submitted by the Author:	06-Jan-2022
Complete List of Authors:	Sania, Ayesha; Columbia University Irving Medical Center, Psychiatry; New York State Psychiatric Institute,<br>Alam, Ahmed; Institute of Epidemiology Disease Control and Research, Virology<br>Alamgir, A. S. M; Institute of Epidemiology Disease Control and Research, Epidemiology; icddr,b, Centre for Food and Waterborne Diseases<br>Andrecka, Joanna; Food and Agriculture Organization of the United Nations<br>Brum, Eric; Food and Agriculture Organization of the United Nations<br>Chadwick, Fergus; University of Glasgow Institute of Biodiversity Animal Health and Comparative Medicine<br>Chowdhury, Tasnuva; University of Glasgow Institute of Biodiversity Animal Health and Comparative Medicine<br>Hasan, Md. Zakiul; Food and Agriculture Organization of the United Nations<br>Hill, Davina; University of Glasgow Institute of Biodiversity Animal Health and Comparative Medicine<br>Khan, Farzana; Institute of Epidemiology Disease Control and Research, Epidemiology<br>Kundegorski, Mikolaj; University of Glasgow Institute of Biodiversity Animal Health and Comparative Medicine<br>Lee, Seonjoo; New York State Psychiatric Institute, Division of Mental Health Data Science; Columbia University Mailman School of Public Health, Department of Biostatistics<br>Rahman , Mahbubur ; Institute of Epidemiology Disease Control and Research, Epidemiology<br>Rayport, Yael; Columbia University Irving Medical Center, Department of Psychiatry; New York State Psychiatric Institute, Neuroscience<br>Shirin, Tahmina; Institute of Epidemiology Disease Control and Research, Virology<br>Tasneem, Motahara; Food and Agriculture Organization of the United Nations<br>Hampson, Katie; University of Glasgow Institute of Biodiversity Animal Health and Comparative Medicine
Keywords:	COVID-19, Diagnostic microbiology < INFECTIOUS DISEASES, Public health < INFECTIOUS DISEASES

I, the Submitting Author has the right to grant and does grant on behalf of all authors of the Work (as defined in the below author licence), an exclusive licence and/or a non-exclusive licence for contributions from authors who are: i) UK Crown employees; ii) where BMJ has agreed a CC-BY licence shall apply, and/or iii) in accordance with the terms applicable for US Federal Government officers or employees acting as part of their official duties; on a worldwide, perpetual, irrevocable, royalty-free basis to BMJ Publishing Group Ltd ("BMJ") its licensees and where the relevant Journal is co-owned by BMJ to the co-owners of the Journal, to publish the Work in this journal and any other BMJ products and to exploit all rights, as set out in our licence.

The Submitting Author accepts and understands that any supply made under these terms is made by BMJ to the Submitting Author unless you are acting as an employee on behalf of your employer or a postgraduate student of an affiliated institution which is paying any applicable article publishing charge ("APC") for Open Access articles. Where the Submitting Author wishes to make the Work available on an Open Access basis (and intends to pay the relevant APC), the terms of reuse of such Open Access shall be governed by a Creative Commons licence – details of these licences and which Creative Commons licence will apply to this Work are set out in our licence referred to above.

Other than as permitted in any relevant BMJ Author's Self Archiving Policies, I confirm this Work has not been accepted for publication elsewhere, is not being considered for publication elsewhere and does not duplicate material already published. I confirm all authors consent to publication of this Work and authorise the granting of this licence.

For peer review only

Rapid Antigen Testing by Community Health Workers to improve detection of SARS-CoV-2 in Dhaka, Bangladesh

Ayesha Sania*, Department of Psychiatry, Columbia University Irving Medical Center, New York, USA

Ahmed Nawsher Alam, Virology, Institute of Epidemiology, Disease Control and Research, Dhaka, Bangladesh

ASM Alamgir, Entomology, Institute of Epidemiology, Disease Control and Research, Dhaka, Bangladesh

Joanna Andrecka, Food and Agriculture Organization of the United Nations, Dhaka, Bangladesh

Eric Brum, Food and Agriculture Organization of the United Nations, Dhaka, Bangladesh

Fergus Chadwick, Institute of Biodiversity Animal Health & Comparative Medicine, University of Glasgow, Glasgow, United Kingdom

Tasnuva Chowdhury, Institute of Biodiversity Animal Health & Comparative Medicine, University of Glasgow, Glasgow, United Kingdom

MD Zakiul Hasan, Food and Agriculture Organization of the United Nations, Dhaka, Bangladesh

Davina L. Hill, Institute of Biodiversity Animal Health & Comparative Medicine, University of Glasgow, Glasgow, United Kingdom

Farzana Khan, Epidemiology, Institute of Epidemiology, Disease Control and Research, Dhaka, Bangladesh

Mikolaj E. Kundegorski, Institute of Biodiversity Animal Health & Comparative Medicine, University of Glasgow, Glasgow, United Kingdom; Food and Agriculture Organization of the United Nations, Dhaka, Bangladesh

Seonjoo Lee, Mental Health Data Science, New York State Psychiatric Institute and Biostatistics and Psychiatry, Columbia University, New York, USA

Mahbubur Rahman, Epidemiology, Institute of Epidemiology, Disease Control and Research, Dhaka, Bangladesh

Yael K. Rayport, Developmental Neuroscience, Columbia University, New York, USA

Tahmina Shirin, Virology, Institute of Epidemiology, Disease Control and Research, Dhaka, Bangladesh

Motahara Tasneem, Food and Agriculture Organization of the United Nations, Dhaka, Bangladesh

For peer review only

Katie Hampson, Institute of Biodiversity Animal Health & Comparative Medicine, University of Glasgow, Glasgow, United Kingdom

Word Count (excluding title page, abstract, references, figures, tables): 3232

***Corresponding author:** Ayesha Sania,

Email: as4823@cumc.columbia.edu

Department of Psychiatry, Columbia University Medical Center 1051 Riverside Drive New York, NY, USA.

For peer review only

ABSTRACT

Objective: To evaluate the diagnostic performance and feasibility of rapid antigen testing for SARS-CoV-2 detection by Community Support Teams in low-income communities.

Setting: Rapid antigen testing was performed by community health workers trained and supervised by medical technicians in low-income communities in Dhaka, Bangladesh**.**

Participants: Between May 19 and July 11, 2021, 1240 nasal and saliva samples were collected from symptomatic individuals and up to two additional household members.

Results: Rapid antigen test performance was calibrated against the gold standard PCR. The sensitivity of rapid antigen tests was 0.68 on nasal samples (95% confidence interval, CI: 0.62-0.73) and 0.41 on saliva (CI: 0.35, 0.46), with specificity also higher on nasal samples (0.98, 95% CI: 0.97-0.99) than saliva (0.87, 95% CI: 0.85, 0.90). Testing two additional household members increased sensitivity to 0.71 on nasal samples (CI: 0.65, 0.76), with a slight drop in specificity (0.96, 95% CI: 0.94, 0.97). Sensitivity on saliva rose to 0.48 (95% CI: 0.42-0.54) with two additional household members tested but remained lower than sensitivity on nasal samples. Testing in these low-income communities has since increased fourfold through mobilization of these community health workers for sample collection.

Conclusions: Rapid antigen testing on nasal swabs can be effectively performed by community health workers yielding equivalent sensitivity and specificity to the literature. Household testing by community health workers in low-resource settings is an inexpensive and scalable approach that can greatly increase capacity, accessibility, and the effectiveness of control measures through immediately actionable results.

Strengths and limitations of this study

- To the best of our knowledge this is the first study to report on the performance of rapid antigen testing at the household level.
- We also report on sensitivity and specificity of the rapid antigen tests when additional household members are tested.
- A limitation of our study is the lack of data on Ct values which precluded evaluation of the performance of rapid antigen tests according to viral load (with Ct values as a proxy).
- The study was conducted in a low-resource community setting among a large sample of subjects (n=1223) with all specimens collected by community health workers.
- Our study provides an example that can be replicated in many community-based programs in Bangladesh and in LMIC communities.

INTRODUCTION

Since the start of the coronavirus (COVID-19) pandemic, countries have sought to develop rapid and accessible approaches to diagnostic testing. The capacity for real-time PCR, the gold standard diagnostic for SARS-CoV-2, has ramped up around the world. Nonetheless, limitations remain in many low-resource settings because of the high cost, relatively slow turnaround time, and need for specialized laboratory setup and highly skilled personnel to perform PCR.(1–4) Rapid antigen tests are a promising alternative, as they are inexpensive, fast to perform (typically returning results within 15 minutes), and require only minimal technical skills and infrastructure.(5) Rapid antigen tests can therefore be used for symptomatic testing and as a screening tool to monitor the epidemiological situation. However, research is needed to evaluate the performance of rapid antigen tests and to develop an effective model for their deployment in many low- and middle-income country (LMIC) settings where adoption has been slow.

The diagnostic accuracy of rapid antigen tests has been evaluated in multiple clinical and laboratory studies, reporting high specificity and moderate to high sensitivity.(6–9) A recent meta-analysis of 133 clinical studies reported sensitivity of 76.3% (95% CI: 73.1% to 79.2%), rising to 95.8% (95% CI: 92.3% to 97.8%) when analyses were restricted to samples with high viral load (i.e., Ct values < 25).(10) When the goal of testing is to mitigate transmission, the ideal test would be one that not just identifies the presence of SARS-CoV-2, but identifies those individuals that contribute to virus transmission. (11) Therefore, despite their lower sensitivity compared to the gold standard, and because of the immediacy of their results, as well as their low cost and ease of administration, rapid antigen tests are an ideal method for use in the community. Importantly, the direct administration of rapid antigen tests at households has the potential to lower testing costs and reduce other logistics barriers and risks, including the movement of infectious individuals when seeking tests. However, questions remain around the most appropriate sample type and delivery models for feasible and effective rapid antigen testing in communities.

Many countries that are constrained by limited diagnostic and healthcare infrastructure and human resources have used community health workers to aid service delivery during the pandemic.(12,13) In June 2020, responding to the Bangladesh Preparedness and Response Plan call to emergency action, the Food and Agriculture Organization of the United Nations, the United Nations Populations Fund, and a collective of partner international agencies, non-governmental and civil society organizations (see acknowledgments) initiated a Community Support Team intervention under the leadership of the Bangladesh Government's Directorate General of Health Services (DGHS). Community Support Teams comprised volunteers trained to search and screen symptomatic COVID-19 cases for preventing symptomatic transmission and were mobilized throughout the two city corporations of Dhaka, with an emphasis on socioeconomically deprived areas. Using this network, we evaluated the diagnostic performance and feasibility of rapid antigen testing for the identification of COVID-19 cases in the

community. Here we report on the sensitivity and specificity of rapid antigen testing by community workers on nasal and saliva samples from symptomatic individuals and their household members, including whether testing multiple household members can improve diagnostic performance. Based on these experiences in Dhaka, we further discuss the potential for community health workers to scale up rapid antigen testing for SARS-CoV-2 in low-income communities.

For peer review only

METHODS

Data Collection:

Community Support Teams comprised local volunteers that were trained by medical technologists to search for and identify suspected COVID-19 cases through hotline calls and community-based channels, and to counsel them in preventative measures, focusing on mask-wearing, isolation for suspected cases, and quarantine of contacts for 14 days, as well as prescribing over-the-counter medications, and in case of severe symptoms refer them to dedicated telemedicine services. At the time of the study, 750 volunteers, hereafter referred to as community health workers, within 465 Community Support Teams were operating across underrepresented vulnerable communities in Dhaka.

Between May 19 and July 11, 2021, study participants (n=1240) were recruited from low-income communities in Dhaka by a subset of community health workers (n=60, split into 30 teams of two persons) who had been trained in sample collection and testing by medical technologists. At this time community health workers were primarily identifying cases through systematic searches within their communities. Participants aged 16 years or older with no previous medical history of bleeding disorders were considered eligible for the study. Participants were suspected to have COVID-19 infection if they reported with a fever (>38℃ or >37.5℃ following use of antipyretics) or a history of fever and had one or more of the following symptoms: difficulty breathing, cough, diarrhea, headache, loss of smell, loss of taste, muscle pain, red eyes, runny nose, sore throat, tiredness, vomiting, or a wet cough. In each household, these suspected COVID-19 cases (index persons) were tested by the community health workers using rapid antigen tests, and then up to two additional household members, regardless of symptoms, were also tested. These additional household members were recruited if they met the eligibility criteria and were available in the household at the time of testing, with those who had most contact with the index cases prioritized for selection. Community health workers uploaded participants' metadata, including their age, gender, and symptoms as well as rapid antigen test results (see below), to a centralized database via a mobile application. Methods are described in further detail by Chadwick et al.(14)

From each participant, nasal swabs from each nostril, as well as two oropharyngeal (OP) swabs, and raw saliva were collected. One nasal and OP swab was pooled for RT-PCR following protocols described by Vogels et al.(15) The swabs were sent to Bangladesh's Institute of Epidemiology, Disease Control, and Research (IEDCR) for PCR testing via cold storage, with samples for PCR testing returned immediately using dedicated transportation. The nasal and saliva samples were tested independently with rapid antigen tests (SD Biosensor STANDARD™ Q COVID-19 Ag Test BioNote) in the household. The manufacturer reports that the STANDARD Q COVID-19 Ag Nasal Home Test has a sensitivity of 94.94% (75/79) and

For peer review only - http://bmjopen.bmj.com/site/about/guidelines.xhtml

specificity of 100% (217/217)(16) and that the STANDARD Q COVID-19 Ag Saliva Home Test has a sensitivity of 94.74% (18/19) and a specificity of 100% (73/73).(17)

All participants provided written informed consent for the health screening, collection of personal information and samples, and test results to be analyzed in the study. The study protocol was approved by the Institutional Review Board at the IEDCR, Ministry of Health, Bangladesh, IEDCR/IRB/2021/04.

Patient involvement

Patients were not involved in setting the research question or the outcomes measures, but patients and their family members were intimately involved in the design and implementation of the study. Patients and their family members received their COVID-19 test results from trusted community health care workers. They were also advised on isolation and quarantine measures, and they were connected with Governments telemedicine services when they needed.

Statistical Analyses:

We assessed the distribution of patient age and sex by the numbers of participating household members using mean (SD) and counts (percentages) for continuous and categorical variables, respectively. We compared group differences using ANOVA and chi-square tests as appropriate. Test sensitivity, specificity, positive predictive values (PPV), and negative predictive values (NPV) were estimated with their 95% exact binomial confidence intervals(18) for all samples and results were further stratified by the numbers of additional household members tested. Based on the estimated rapid antigen test sensitivity and reduced cost of rapid testing compared to laboratory testing using PCR we also examined the potential for improved case detection.

All analyses were undertaken in R Statistical Software (v4.0.02)(19) and EpiR R package (v2.0.39).(20)

For peer review only

RESULTS

During the study period, 1240 subjects consented to participate in the study. Of these subjects, 17 were excluded from the analyses due to missing information on the clinical definition used to diagnose a suspected case of COVID-19 (N=9, 0.7%), invalid results from rapid antigen tests on saliva samples or nasal swabs (4, 0.3%), or missing or invalid PCR results (N=4, 0.3%). Table 1 shows the sex and age distribution of the participants by index person (N=1223), first additional household member (N=710), and second additional household member (N=283, 23%)

Table1. Demographic Information of the study participants.

		Index person **(N=1223)**	**Second household member (N=710)**	**Third household member (N=283)**	**Total (N=2216)**
**sex***	female	575 (47.0%)	397 (55.9%)	151 (53.4%)	1123 (50.7%)
	male	640 (52.3%)	312 (43.9%)	132 (46.6%)	1084 (48.9%)
**age***	Mean (SD)	36.522 (14.265)	36.784 (14.417)	34.735 (15.434)	36.377 (14.476)

* participants had missing values.

Rapid antigen tests performed better on nasal than saliva samples: The sensitivity, specificity, positive predictive value (PPV), and negative predictive value (NPV) of rapid antigen tests on nasal swabs and saliva samples are shown in Table 2 and Figure 1 (red bars). Of all subjects with complete test results (N=1223), 291 were PCR positive. Of these, 198 were rapid antigen test positive by nasal swab and 118 were rapid antigen test positive by saliva sample. Therefore, the overall sensitivity of rapid antigen testing on nasal samples was 0.68 (95% confidence interval, CI: 0.62-0.73) and on saliva samples was 0.41 (95% CI: 0.35-0.46). Saliva samples picked up some PCR-positive subjects who were not positive in the nasal swab (n = 22), and vice versa (n = 102). Specificity of nasal swabs (0.98, 95% CI: 0.97-0.99) was also higher than saliva samples (0.87, 95% CI: 0.85-0.90). The overall accuracy of rapid antigen tests on nasal swabs was higher (0.91 vs 0.76) compared to accuracy on saliva samples (table 2).

For peer review

Table 2. Performance of rapid antigen tests compared to PCR, among all household members tested. The total sample size was 1223 index subjects, 95% confidence intervals are shown in brackets. RAT = rapid antigen test, PPV = positive predictive value, NPV = negative predictive value.

	**Nasal sample**	**Saliva sample**
	PCR + PCR - Total<br>RAT + 198 21 219<br>RAT - 93 911 1004<br>Total 291 932 1223	PCR + PCR - Total<br>RAT + 118 117 235<br>RAT - 173 815 988<br>Total 291 932 1223
Sensitivity	0.68 (0.62,0.73)	0.41 (0.35, 0.46)
Specificity	0.98 (0.9, 0.99)	0.87 (0.85, 0.90)
PPV	0.90 (0.86, 0.94)	0.50 (0.44, 0.57)
NPV	0.91 (0.89, 0.92)	0.82 (0.80, 0.85)
Accuracy	0.91 (0.89, 0.92)	0.76 (0.74, 0.79)

The probability of a false negative rapid antigen test (i.e. discordant with a positive PCR test) increased with the number of days between self-reported symptom onset and testing, and very few false negatives occurred within the first three days of symptoms (Figure 2). However, our sample size (55 false negative rapid antigen tests from 736 test results with self-reported symptoms onset date) was too small for rigorous quantitative analysis of this relationship.

Testing additional household members yields better rapid antigen test performance: The sensitivity of rapid antigen tests on nasal swabs increased from 0.68 (95% CI: 0.62-0.73) when only the index person was tested, to 0.70 (95% CI: 0.65-0.76) when an additional household member was tested (Figure 1, green bars) and to 0.71 (95% CI: 0.65-0.76) when a second household member was also tested (Figure 1, blue bars), although it should be noted that only a subset of households had additional members available for testing at the time of community health worker visits. With this gain in sensitivity, there was a corresponding drop in

For peer review only - http://bmjopen.bmj.com/site/about/guidelines.xhtml

specificity with additional household members, from 0.98 (95% CI: 0.97-099) to 0.96 (95% CI: 0.94-0.97) for two additional members tested (Figure 1). Similarly, sensitivity of rapid antigen testing on saliva samples increased from 0.42 (95% CI: 0.35-0.46) to 0.48 (95% CI: 0.42-0.54) with two additional household members tested, although there was a corresponding drop in specificity from 0.87 (95%CI: 0.85-0.90) to 0.82 (95% CI: 0.79-0.84). Testing both sample types yielded a modest increase in sensitivity but a large drop in specificity (Figure 1).

Community health workers achieved higher testing coverage than routine laboratory testing coverage: During the validation phase of this study between May 19, 2021, and July 11, 2021, community health workers collected 73% of the samples from these low-income communities that were tested at IEDCR (Figure 3). Using the rapid antigen testing approach, whereby individuals were tested in their households and immediately given their results, willingness to be tested greatly increased. Moreover, shortly after the study began, the Delta variant swept across Bangladesh, and by late June community workers were collecting around 90% of samples in these communities as case numbers surged. The community health workers trained to collect samples for this study continued to collect samples for IEDCR as part of their routine PCR surveillance during the third wave of infection (Figure 3). This period of implementation demonstrated that community health workers can collect and test over 4 times more households compared to the standard approach of community testing (Figure 3), which relies upon follow-up visits by medical technologists the day after symptomatic households are identified by community health workers. Assuming a constrained diagnostic testing budget and a cost of \$5 per rapid diagnostic test and of \$30 per PCR laboratory diagnosis, we calculate that household (rapid antigen) testing would detect four times more cases than laboratory (PCR) testing, despite its lower sensitivity, under a range of prevalence levels (from <0.05 to >0.4).

For peer review only - http://bmjopen.bmj.com/site/about/guidelines.xhtml

DISCUSSION:

We evaluated the performance of rapid antigen testing on nasal and saliva samples collected by community health workers at the household level in low-income communities across Dhaka, Bangladesh. We estimated that rapid antigen tests on nasal swabs had moderate sensitivity and high specificity as expected in a community setting and found that testing additional household members increased the sensitivity of detection of COVID-19 infection without compromising specificity by much. This study capitalized on a network of community volunteers who were initially trained to clinically assess individuals reporting COVID-19 symptoms as part of the crisis response and demonstrated that community health workers can administer rapid antigen tests effectively and achieve high coverage in community settings. Our results support recommendations for scaling up rapid testing as part of the COVID-19 response in low-resource settings.

Our report of the superior performance of rapid antigen tests on nasal samples compared to saliva samples confirms the existing literature from clinical and laboratory settings.(21) A recent study reported poor concordance between nasopharyngeal swab specimens and saliva samples (75.9%, kappa coefficient 0.310).(22) However, a meta-analysis examining the performance of PCR concluded that saliva samples were as sensitive as nasopharyngeal swabs.(23,24) A likely explanation for the lower sensitivity of rapid antigen tests on saliva could be due to lower viral load, which would have less impact with PCR.(25) A study on the diagnostic accuracy of rapid antigen tests conducted at testing sites in the Netherlands found that their sensitivity increased from 60% to 85% after applying a viral load cut-off as a proxy for infectiousness.(26) The performance of rapid antigen tests further depends on the virus variant, with a higher viral load reported in saliva for the Beta variant compared to others.(27) Although saliva samples have advantages including easier administration (typically self-collected for testing in many high-income settings), being less invasive, and causing minimal discomfort,(28) we confirm the superiority of nasal swabs in terms of sensitivity and specificity, at least for the Delta variant, which was most prevalent during our study.(29)

Our estimate of rapid antigen test sensitivity on nasal samples (68%) is comparable to that reported by Cerruti et al. (70.6 %) from a mix of symptomatic and asymptomatic participants.(30) Estimates of sensitivity reported by the manufacturer and by studies conducted among symptomatic patients at the point of care are, however, much higher than in our report. SD biosensor, the manufacturer of the STANDARD Q COVID-19 Ag Nasal Home Test reports a sensitivity of 94.94% (16), and an SD biosensor rapid antigen test had a sensitivity of 89.0% at a point-of-care community-based testing center, where the vast majority of the patients were symptomatic (97.8%).(31) Higher sensitivity in laboratory and clinical settings is likely due to higher viral loads. Several prior reports including a recent meta-analysis confirmed that rapid antigen tests have their highest sensitivity in samples with high viral load (Ct value <25)(10) and therefore lower sensitivity is to be expected when testing in the community includes both

For peer review only

individuals who are currently infectious as well as those who are recovering (Figure 2).(11) Moreover, a point-of-care rapid antigen test study found that when rapid antigen tests and PCR results were discordant, SARS-CoV-2 could not be cultured from the samples, suggesting that these patients were unlikely to be infectious.(32) Thus, from a public health perspective, where curtailing disease transmission is the goal, only people with high viral loads need to be detected as they are capable of transmitting the disease to others.(4,33) Rapid antigen tests are better suited to screening for people with high viral loads, the population which poses the greatest risk for the community. This combined with their reduced costs, logistics, and speed of turnaround makes them advantageous for supporting the rapid and effective implementation of targeted prevention measures.

To the best of our knowledge, this is the first study to report on the performance of rapid antigen testing at the household level. Our reports of higher sensitivity when testing additional household members is similar to the interpretation of flock-based testing in animals.(34) For highly pathogenic avian influenza H5N1 in backyard poultry flocks, Robyn et al.(35) demonstrated that combining a standard case definition with one or more rapid antigen tests can increase the sensitivity of case detection to closer to that of the gold standard PCR while maintaining specificity. Importantly, our study was conducted in a low-resource community setting among a large sample of subjects (n=1223) with all specimens collected by community health workers. A limitation of our study is the lack of data on Ct values which precluded evaluation of the performance of rapid antigen tests according to viral load (with Ct values as a proxy). The higher sensitivity and specificity in samples with high viral load have been confirmed in several prior studies.(4,11)

In our study, community health workers collected and tested samples from individuals in low-income households in Dhaka. These communities are underserved by routine testing procedures in Bangladesh, which require that individuals visit a testing facility and pay for a test (USD15-50 for a PCR with results returned 2-5 days later and more recently USD 5-10 for a rapid antigen test). To access care for complications due to COVID-19 at peak times during the pandemic, patients were required to present a positive test result, whereas those requiring non-COVID-19 care were required to present with a negative test, affecting the care that individuals could access. Community health workers were therefore able to greatly increase the number of people who were tested from these communities. Moreover, people in these communities were keen to be tested especially as results were returned immediately in the privacy of their own household. That community health workers were known and trusted in these communities likely contributed to people's willingness to be tested. During the rapid antigen testing validation, the Delta variant began to surge in Dhaka, and community health workers trained to collect samples as part of this study were deployed to continue sample collection for PCR testing. In August 2021 Community Support Teams supported by FAO and UNFPA collected over 80% of the samples from these communities that were tested at the national laboratory, IEDCR, during Bangladesh's third wave,

For peer review only

which was the largest peak experienced so far. Sample collection by community health workers proved more efficient and acceptable in these communities and meant that IEDCR was better able to meet testing demand at this critical time. Had rapid tests been available, the impacts would have likely been greater.

Rapid tests are increasingly being deployed in community settings for infectious disease control. In Tanzania, community health workers successfully used rapid tests for the detection of malaria, conducted early fever case management, and made treatment more accessible to rural populations.(36) Community health workers have likewise been deployed for infectious disease control, in response to outbreaks of SARS, swine flu, Ebola, rabies, and MERS-CoV. During the COVID-19 pandemic, many LMIC governments have established community health worker programs to support diagnostic testing, treatment, immunization, and contact tracing.(37) Global initiatives have sought to reduce the costs of rapid antigen tests for COVID-19 to below US$5 each, with catalytic funding to guarantee prices for LMICs.(12, 26) Our study demonstrates that community health workers are capable of effective household-level rapid antigen testing in low-income communities in Dhaka, providing an example that can be replicated in many community-based programs in Bangladesh and proof-of-principle for the scalability of rapid testing to mitigate COVID-19 in LMIC communities generally.

For peer review only

Funding: The Bill and Melinda Gates Foundation (BMGF) funded work by FAO (INV-022851) and UoG reports funding from Wellcome (095787/Z/11/Z).

Acknowledgments: The authors thank the Directorate General of Health Services (DGHS), Bangladesh, and the Institute of Epidemiology Disease Control and Research (IEDCR) for ongoing SAR-CoV-2 surveillance and prevention measures. The Community Support Team intervention is funded by the World Bank, USAID, and FCDO (UKAID). This multi-sectoral, collaborative initiative is led by the DGHS with technical and operational support from several UN Organizations (FAO, UNFPA, UNICEF, WFP), with community health workers and volunteers provided by NGOs (BRAC) and civil society organizations (Youth Platform, Utshargo Foundation, Himu Paribahan, CDP, Platform and Young Bangla). Additional technical support was provided by national institutions such as a2i and icddr,b. We are extremely grateful to the community support team workers who have volunteered throughout this emergency, dedicating time and energy to provide support and response in these communities and without whom this study would not have been possible, as well as to the Zonal Executive Officers, Ward Councilors, Chief Health Officers, Deputy Chief Health Officers and other administrative members of Dhaka North and Dhaka South City Corporations. The University of Glasgow LMICs COVID-19 working group provided valuable feedback and discussion.

Author contributions: Author Contributions: EB, KH, and AS had full access to all of the data in the study and took responsibility for the integrity of the data and the accuracy of the data analysis. EB, AS, and KH designed the study, and EB secured funding. AS, EB, TS, ASMA, MR, ANA, FK, and KH planned the study and provided scientific, administrative, and technical support for its implementation. TC developed the standard operating procedures for sampling. MEK developed the data collection tools and managed the data. JA coordinated activities. MT led the field implementation, and with ZH trained and supervised the community health workers and provided technical support. AS wrote the data analysis plan, and the first manuscript draft. SL conducted data analysis and revised the manuscript. YKR assisted in literature reviews and manuscript drafting. KH, FC, and DH undertook the data analysis and manuscript revisions. All authors reviewed and approved the final manuscript.

Data availability: Data and code for replication of the study results available: https://github.com/boydorr/BGD_CST_rapid_testing

Ethics approval

This study was approved by the Institutional Review Board at the IEDCR, Ministry of Health, Bangladesh, IEDCR/IRB/2021/04.

Competing interests: None declared.

For peer review only

REFERENCES

1 Brihn A, Chang J, OYong K, et al. Diagnostic Performance of an Antigen Test with RT-PCR for the Detection of SARS-CoV-2 in a Hospital Setting - Los Angeles County, California, June-August 2020. MMWR Morb Mortal Wkly Rep. 2021 May 14;70(19):702–6.
2. Corman VM, Landt O, Kaiser M, et al. Detection of 2019 novel coronavirus (2019-nCoV) by real-time RT-PCR. Eurosurveillance. 2020 Jan 23;25(3):2000045.
3. Kucirka LM, Lauer SA, Laeyendecker O, et al. Variation in False-Negative Rate of Reverse Transcriptase Polymerase Chain Reaction–Based SARS-CoV-2 Tests by Time Since Exposure. Ann Intern Med. 2020 May 13;M20-1495.
4. Larremore DB, Wilder B, Lester E, et al. Test sensitivity is secondary to frequency and turnaround time for COVID-19 screening. Sci Adv. 2021 Jan;7(1):eabd5393.
5. World Health Organization. Antigen-detection in the diagnosis of SARS-CoV-2 infection using rapid immunoassays: Interim guidance. 2020 Sep [cited 2021 Sep 22]. Available: https://www.who.int/publications-detail-redirect/antigen-detection-in-the-diagnosis-of-sars-cov-2infection-using-rapid-immunoassays
6. Krüttgen A, Cornelissen CG, Dreher M, et al. Comparison of the SARS-CoV-2 Rapid antigen test to the real star Sars-CoV-2 RT PCR kit. J Virol Methods. 2021 Feb;288:114024.
7. Hirotsu Y, Maejima M, Shibusawa M, et al. Comparison of automated SARS-CoV-2 antigen test for COVID-19 infection with quantitative RT-PCR using 313 nasopharyngeal swabs, including from seven serially followed patients. Int J Infect Dis IJID Off Publ Int Soc Infect Dis. 2020 Oct;99:397–402.
8. Pilarowski G, Lebel P, Sunshine S, et al. Performance Characteristics of a Rapid Severe Acute Respiratory Syndrome Coronavirus 2 Antigen Detection Assay at a Public Plaza Testing Site in San Francisco. J Infect Dis. 2021 Jan 4;223(7):1139–44.
9. Pray IW. Performance of an Antigen-Based Test for Asymptomatic and Symptomatic SARS-CoV-2 Testing at Two University Campuses — Wisconsin, September–October 2020. MMWR Morb Mortal Wkly RepAvailable. 2021 [cited 2021 Oct 4];69. Available: https://www.cdc.gov/mmwr/volumes/69/wr/mm695152a3.htm
10. Brümmer LE, Katzenschlager S, Gaeddert M, et al. Accuracy of novel antigen rapid diagnostics for SARS-CoV-2: A living systematic review and meta-analysis. PLOS Med. 2021 Aug 12;18(8):e1003735.
11. Petersen I, Crozier A, Buchan I, et al. Recalibrating SARS-CoV-2 Antigen Rapid Lateral Flow Test Relative Sensitivity from Validation Studies to Absolute Sensitivity for Indicating Individuals Shedding Transmissible Virus. Clin Epidemiol. 2021 Oct 14;13:935–40.
12. David N, Mash R. Community-based screening and testing for Coronavirus in Cape Town, South Africa: Short report. Afr J Prim Health Care Fam Med. 2020 Jun 3;12(1):3.
13. Bezbaruah S, Wallace P, Zakoji M, et al. Roles of community health workers in advancing health security and resilient health systems: emerging lessons from the COVID-19 response in the South-East Asia Region. WHO South-East Asia J Public Health. 2021 Feb 1;10(3):41.
14. Chadwick F, Clark J, Chowdhury S, et al. Combining Rapid Antigen Testing and Syndromic Surveillance Improves Sensitivity and Specificity of COVID-19 Detection: A Community-Based Prospective Diagnostic StudyAvailable. Rochester, NY: Social Science Research Network; 2021 Sep [cited 2021 Oct 4]. Report No.: ID 3927057.
15. Vogels CBF, Brito AF, Wyllie AL, et al. Analytical sensitivity and efficiency comparisons of SARS-CoV-2 RT–qPCR primer–probe sets. Nat Microbiol. 2020 Oct;5(10):1299–305.
16. SD BIOSENSOR | PRODUCTS. Available. [cited 2021 Oct 4]. Available:

For peer review only

https://www.sdbiosensor.com/product/main?bcode=11&bscode=COVID-19%20Products
17. SD BIOSENSOR | PRODUCTS. Available. [cited 2021 Oct 14]. Available: https://www.sdbiosensor.com/product/product_view?product_no=236
18. Collett D. Modelling Binary Data. Boca Raton Florida: Chapman and Hall/CRC; 2002. 24 p.
19. R Core Team. R: A language and environment for statistical computing. R Foundation for Statistical Computing. Vienna, Austria; 2021.
20. Mark Stevenson, Sergeant E. epiR: Tools for the Analysis of Epidemiological Data. 2021.
21. Nagura-Ikeda M, Imai K, Tabata S, et al. Clinical Evaluation of Self-Collected Saliva by Quantitative Reverse Transcription-PCR (RT-qPCR), Direct RT-qPCR, Reverse Transcription-Loop-Mediated Isothermal Amplification, and a Rapid Antigen Test To Diagnose COVID-19. J Clin Microbiol. 2020 Aug 24;58(9):e01438-20.
22. Uwamino Y, Nagata M, Aoki W, et al. Accuracy of rapid antigen detection test for nasopharyngeal swab specimens and saliva samples in comparison with RT-PCR and viral culture for SARS-CoV-2 detection. J Infect Chemother. 2021 Jul;27(7):1058–62.
23. Lee RA, Herigon JC, Benedetti A, et al. Performance of Saliva, Oropharyngeal Swabs, and Nasal Swabs for SARS-CoV-2 Molecular Detection: a Systematic Review and Meta-analysis. J Clin Microbiol. 2021 Apr 20;59(5):e02881-20.
24. Bastos ML, Perlman-Arrow S, Menzies D, et al. The Sensitivity and Costs of Testing for SARS-CoV-2 Infection With Saliva Versus Nasopharyngeal Swabs : A Systematic Review and Meta-analysis. Ann Intern Med. 2021 Apr;174(4):501–10.
25. Procop GW, Shrestha NK, Vogel S, et al. A Direct Comparison of Enhanced Saliva to Nasopharyngeal Swab for the Detection of SARS-CoV-2 in Symptomatic Patients. J Clin Microbiol. 2020 Oct 21;58(11):e01946-20.
26. Schuit E, Veldhuijzen IK, Venekamp RP, et al. Diagnostic accuracy of rapid antigen tests in asymptomatic and presymptomatic close contacts of individuals with confirmed SARS-CoV-2 infection: cross sectional study. BMJ. 2021 Jul 27;374:n1676.
27. Chu CY, Marais G, Opperman C, et al. Improved performance of saliva for the detection of the B.1.351 variant in South AfricaAvailable. 2021 Mar [cited 2021 Oct 19] p. 2021.03.29.21254563. Available: https://www.medrxiv.org/content/10.1101/2021.03.29.21254563v1
28. Hung K-F, Sun Y-C, Chen B-H, et al. New COVID-19 saliva-based test: How good is it compared with the current nasopharyngeal or throat swab test? J Chin Med Assoc JCMA. 2020 Oct;83(10):891–4.
29. Saha S, Tanmoy AM, Tanni AA, et al. New waves, new variants, old inequity: a continuing COVID-19 crisis. BMJ Glob Health. 2021 Aug;6(8):e007031.
30. Cerutti F, Burdino E, Milia MG, et al. Urgent need of rapid tests for SARS CoV-2 antigen detection: Evaluation of the SD-Biosensor antigen test for SARS-CoV-2. J Clin Virol. 2020 Nov;132:104654.
31. Berger A, Nsoga MTN, Perez-Rodriguez FJ, et al. Diagnostic accuracy of two commercial SARS-CoV-2 antigen-detecting rapid tests at the point of care in community-based testing centers. PLOS ONE. 2021 Mar 31;16(3):e0248921.
32. Torres I, Poujois S, Albert E, et al. Evaluation of a rapid antigen test (PanbioTM COVID-19 Ag rapid test device) for SARS-CoV-2 detection in asymptomatic close contacts of COVID-19 patients. Clin Microbiol Infect. 2021 Apr;27(4):636.e1-636.e4.
33. Marks M, Millat-Martinez P, Ouchi D, et al. Transmission of COVID-19 in 282 clusters in Catalonia, Spain: a cohort study. Lancet Infect Dis. 2021;629–36.
34. Martin SW, Shoukri M, Thorburn MA. Evaluating the health status of herds based on tests applied to individuals. Prev Vet Med. 1992 Oct 1;14(1):33–43.
35. Robyn M, Priyono WB, Kim LM, et al. Diagnostic sensitivity and specificity of a

For peer review only

participatory disease surveillance method for highly pathogenic avian influenza in household chicken flocks in Indonesia. Avian Dis. 2012 Jun;56(2):377–80.
36. Mubi M, Janson A, Warsame M, et al. Malaria Rapid Testing by Community Health Workers Is Effective and Safe for Targeting Malaria Treatment: Randomised Cross-Over Trial in Tanzania. PLOS ONE. 2011 Jul 5;6(7):e19753.
37. Bhaumik S, Moola S, Tyagi J , et al. Community health workers for pandemic response: a rapid evidence synthesis. BMJ Glob Health. 2020 Jun 10;5(6):e002769.

For peer review only

Figure Legends:

Figure 1. Sensitivity (left) and specificity (right) of rapid antigen testing (RAT) compared to PCR, when 1 (index person), 2 or 3 members of the same household are tested.

Figure 2. The percentage of false negative rapid antigen test results given a positive PCR increases with days from self-reported symptom onset.

Figure 3. IEDCR sample collection and testing in Dhaka from May to September 2021. Routine sample collection by IEDCR (Institute of Epidemiology, Disease Control, and Research) from low-income communities in Dhaka where the Community Support Teams operate. Sample collections by medical technologists are shown in grey (MT). The period of rapid antigen testing is indicated, with samples collected for rapid testing in households shown in red (RAT). The subsequent collection of samples by community health workers for PCR testing during the surge of infection driven by the Delta variant is shown in orange (CST). A marked dip in testing in late July corresponded to a religious festival (Eid Al-Adha).

only

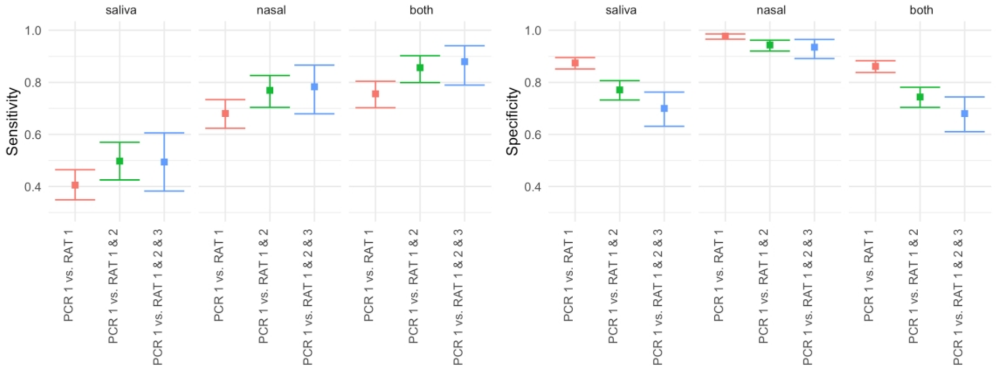

Figure 1. Sensitivity (left) and specificity (right) of rapid antigen testing (RAT) compared to PCR, when 1 (index person), 2 or 3 members of the same household are tested.

696x278mm (118 x 118 DPI)

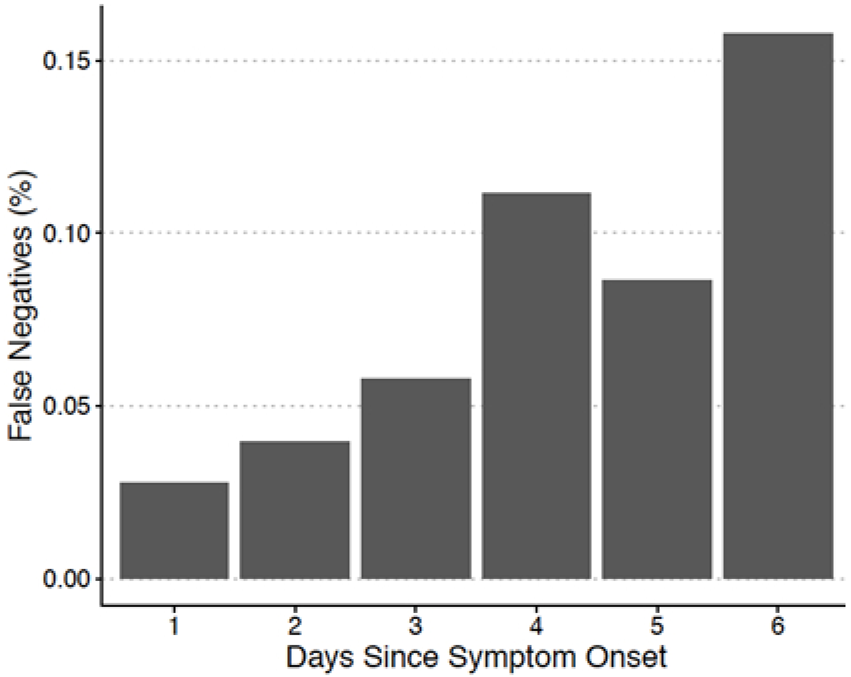

Figure 2. The percentage of false negative rapid antigen test results given a positive PCR increases with days from self-reported symptom onset.

281x226mm (38 x 38 DPI)

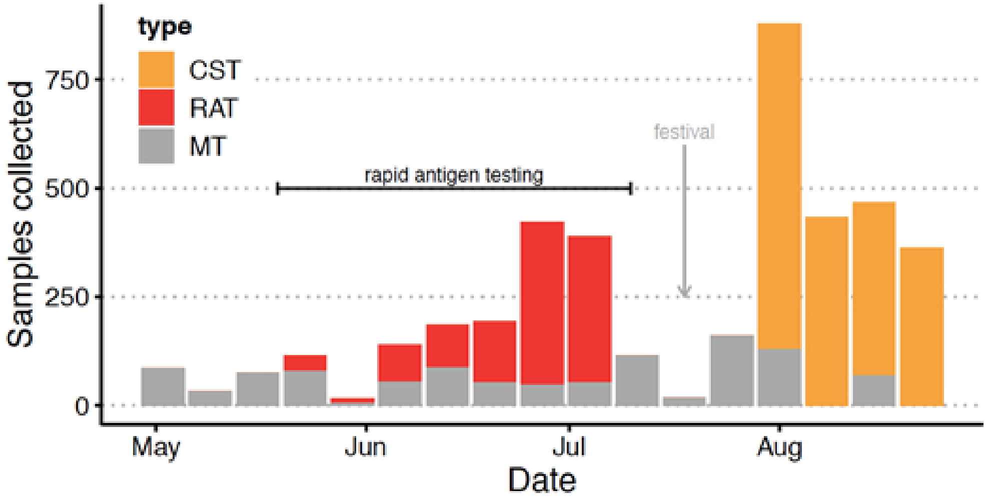

Figure 3. IEDCR sample collection and testing in Dhaka from May to September 2021. Routine sample collection by IEDCR (Institute of Epidemiology, Disease Control, and Research) from low-income communities in Dhaka where the Community Support Teams operate. Sample collections by medical technologists are shown in grey (MT). The period of rapid antigen testing is indicated, with samples collected for rapid testing in households shown in red (RAT). The subsequent collection of samples by community health workers for PCR testing during the surge of infection driven by the Delta variant is shown in orange (CST). A marked dip in testing in late July corresponded to a religious festival (Eid Al-Adha).

352x198mm (38 x 38 DPI)

STROBE Statement—checklist of items that should be included in reports of observational studies

	Item No	Recommendation	Page No
**Title and abstract**	1	(*a*) Indicate the study's design with a commonly used term in the title or the abstract	4
		(*b*) Provide in the abstract an informative and balanced summary of what was done and what was found	4
**Introduction**			
Background/rationale	2	Explain the scientific background and rationale for the investigation being reported	5
Objectives	3	State specific objectives, including any prespecified hypotheses	6
**Methods**			
Study design	4	Present key elements of study design early in the paper	7
Setting	5	Describe the setting, locations, and relevant dates, including periods of recruitment, exposure, follow-up, and data collection	7
Participants	6	(*a*) *Cohort study*—Give the eligibility criteria, and the sources and methods of selection of participants. Describe methods of follow-up<br>*Case-control study*—Give the eligibility criteria, and the sources and methods of case ascertainment and control selection. Give the rationale for the choice of cases and controls<br>*Cross-sectional study*—Give the eligibility criteria, and the sources and methods of selection of participants	7
		(*b*) *Cohort study*—For matched studies, give matching criteria and number of exposed and unexposed<br>*Case-control study*—For matched studies, give matching criteria and the number of controls per case	
Variables	7	Clearly define all outcomes, exposures, predictors, potential confounders, and effect modifiers. Give diagnostic criteria, if applicable	7, 8
Data sources/ measurement	8*	For each variable of interest, give sources of data and details of methods of assessment (measurement). Describe comparability of assessment methods if there is more than one group	*7,8*
Bias	9	Describe any efforts to address potential sources of bias	13
Study size	10	Explain how the study size was arrived at	9
Quantitative variables	11	Explain how quantitative variables were handled in the analyses. If applicable, describe which groupings were chosen and why	8
Statistical methods	12	(*a*) Describe all statistical methods, including those used to control for confounding	8
		(*b*) Describe any methods used to examine subgroups and interactions	8
		(*c*) Explain how missing data were addressed	
		(*d*) *Cohort study*—If applicable, explain how loss to follow-up was addressed<br>*Case-control study*—If applicable, explain how matching of cases and controls was addressed<br>*Cross-sectional study*—If applicable, describe analytical methods taking account of sampling strategy	8
		(*e*) Describe any sensitivity analyses	

Continued on next page

For peer review only - http://bmjopen.bmj.com/site/about/guidelines.xhtml

**Results**			
Participants	13*	(a) Report numbers of individuals at each stage of study—eg numbers potentially eligible, examined for eligibility, confirmed eligible, included in the study, completing follow-up, and analysed	9
		(b) Give reasons for non-participation at each stage	
		(c) Consider use of a flow diagram	
Descriptive data	14*	(a) Give characteristics of study participants (eg demographic, clinical, social) and information on exposures and potential confounders	9
		(b) Indicate number of participants with missing data for each variable of interest	
		(c) *Cohort study*—Summarise follow-up time (eg, average and total amount)	
Outcome data	15*	*Cohort study*—Report numbers of outcome events or summary measures over time	*9*
		Case-control study—Report numbers in each exposure category, or summary measures of exposure	
		Cross-sectional study—Report numbers of outcome events or summary measures	
Main results	16	(*a*) Give unadjusted estimates and, if applicable, confounder-adjusted estimates and their precision (eg, 95% confidence interval). Make clear which confounders were adjusted for and why they were included	10
		(*b*) Report category boundaries when continuous variables were categorized	
		(*c*) If relevant, consider translating estimates of relative risk into absolute risk for a meaningful time period	
Other analyses	17	Report other analyses done—eg analyses of subgroups and interactions, and sensitivity analyses	
**Discussion**			
Key results	18	Summarise key results with reference to study objectives	12
Limitations	19	Discuss limitations of the study, taking into account sources of potential bias or imprecision. Discuss both direction and magnitude of any potential bias	13
Interpretation	20	Give a cautious overall interpretation of results considering objectives, limitations, multiplicity of analyses, results from similar studies, and other relevant evidence	13
Generalisability	21	Discuss the generalisability (external validity) of the study results	13
**Other information**			
Funding	22	Give the source of funding and the role of the funders for the present study and, if applicable, for the original study on which the present article is based	15

*Give information separately for cases and controls in case-control studies and, if applicable, for exposed and unexposed groups in cohort and cross-sectional studies.

Note: An Explanation and Elaboration article discusses each checklist item and gives methodological background and published examples of transparent reporting. The STROBE checklist is best used in conjunction with this article (freely available on the Web sites of PLoS Medicine at http://www.plosmedicine.org/, Annals of Internal Medicine at http://www.annals.org/, and Epidemiology at http://www.epidem.com/). Information on the STROBE Initiative is available at www.strobe-statement.org.

For peer review only

BMJ Open

Rapid antigen testing by community health workers for detection of SARS-CoV-2 in Dhaka, Bangladesh: a cross-sectional study

Journal:	*BMJ Open*
Manuscript ID	bmjopen-2022-060832.R1
Article Type:	Original research
Date Submitted by the Author:	18-Apr-2022
Complete List of Authors:	Sania, Ayesha; Columbia University Irving Medical Center, Psychiatry; New York State Psychiatric Institute,<br>Alam, Ahmed; Institute of Epidemiology Disease Control and Research, Virology<br>Alamgir, A. S. M; Institute of Epidemiology Disease Control and Research, Epidemiology; icddr,b, Centre for Food and Waterborne Diseases<br>Andrecka, Joanna; Food and Agriculture Organization of the United Nations<br>Brum, Eric; Food and Agriculture Organization of the United Nations<br>Chadwick, Fergus; University of Glasgow Institute of Biodiversity Animal Health and Comparative Medicine<br>Chowdhury, Tasnuva; University of Glasgow Institute of Biodiversity Animal Health and Comparative Medicine<br>Hasan, Md. Zakiul; Food and Agriculture Organization of the United Nations<br>Hill, Davina; University of Glasgow Institute of Biodiversity Animal Health and Comparative Medicine<br>Khan, Farzana; Institute of Epidemiology Disease Control and Research, Epidemiology<br>Kundegorski, Mikolaj; University of Glasgow Institute of Biodiversity Animal Health and Comparative Medicine<br>Lee, Seonjoo; New York State Psychiatric Institute, Division of Mental Health Data Science; Columbia University Mailman School of Public Health, Department of Biostatistics<br>Rahman , Mahbubur ; Institute of Epidemiology Disease Control and Research, Epidemiology<br>Rayport, Yael; Columbia University Irving Medical Center, Department of Psychiatry; New York State Psychiatric Institute, Neuroscience<br>Shirin, Tahmina; Institute of Epidemiology Disease Control and Research, Virology<br>Tasneem, Motahara; Food and Agriculture Organization of the United Nations<br>Hampson, Katie; University of Glasgow Institute of Biodiversity Animal Health and Comparative Medicine
**Primary Subject Heading**:	Global health

Secondary Subject Heading:	Epidemiology, Infectious diseases
Keywords:	COVID-19, Diagnostic microbiology < INFECTIOUS DISEASES, Public health < INFECTIOUS DISEASES

SCHOLARONE™ Manuscripts

I, the Submitting Author has the right to grant and does grant on behalf of all authors of the Work (as defined in the below author licence), an exclusive licence and/or a non-exclusive licence for contributions from authors who are: i) UK Crown employees; ii) where BMJ has agreed a CC-BY licence shall apply, and/or iii) in accordance with the terms applicable for US Federal Government officers or employees acting as part of their official duties; on a worldwide, perpetual, irrevocable, royalty-free basis to BMJ Publishing Group Ltd ("BMJ") its licensees and where the relevant Journal is co-owned by BMJ to the co-owners of the Journal, to publish the Work in this journal and any other BMJ products and to exploit all rights, as set out in our licence.

The Submitting Author accepts and understands that any supply made under these terms is made by BMJ to the Submitting Author unless you are acting as an employee on behalf of your employer or a postgraduate student of an affiliated institution which is paying any applicable article publishing charge ("APC") for Open Access articles. Where the Submitting Author wishes to make the Work available on an Open Access basis (and intends to pay the relevant APC), the terms of reuse of such Open Access shall be governed by a Creative Commons licence – details of these licences and which Creative Commons licence will apply to this Work are set out in our licence referred to above.

Other than as permitted in any relevant BMJ Author's Self Archiving Policies, I confirm this Work has not been accepted for publication elsewhere, is not being considered for publication elsewhere and does not duplicate material already published. I confirm all authors consent to publication of this Work and authorise the granting of this licence.

For peer review only

Rapid antigen testing by community health workers for detection of SARS-CoV-2 in Dhaka, Bangladesh: a cross-sectional study

Ayesha Sania*, Department of Psychiatry, Columbia University Irving Medical Center, New York, USA

Ahmed Nawsher Alam, Virology, Institute of Epidemiology, Disease Control and Research, Dhaka, Bangladesh

ASM Alamgir, Entomology, Institute of Epidemiology, Disease Control and Research, Dhaka, Bangladesh

Joanna Andrecka, Food and Agriculture Organization of the United Nations, Dhaka, Bangladesh

Eric Brum, Food and Agriculture Organization of the United Nations, Dhaka, Bangladesh

Fergus Chadwick, Institute of Biodiversity Animal Health & Comparative Medicine, University of Glasgow, Glasgow, United Kingdom

Tasnuva Chowdhury, Institute of Biodiversity Animal Health & Comparative Medicine, University of Glasgow, Glasgow, United Kingdom

MD Zakiul Hasan, Food and Agriculture Organization of the United Nations, Dhaka, Bangladesh

Davina L. Hill, Institute of Biodiversity Animal Health & Comparative Medicine, University of Glasgow, Glasgow, United Kingdom

Farzana Khan, Epidemiology, Institute of Epidemiology, Disease Control and Research, Dhaka, Bangladesh

Mikolaj E. Kundegorski, Institute of Biodiversity Animal Health & Comparative Medicine, University of Glasgow, Glasgow, United Kingdom; Food and Agriculture Organization of the United Nations, Dhaka, Bangladesh

Seonjoo Lee, Mental Health Data Science, New York State Psychiatric Institute and Biostatistics and Psychiatry, Columbia University, New York, USA

Mahbubur Rahman, Epidemiology, Institute of Epidemiology, Disease Control and Research, Dhaka, Bangladesh

Yael K. Rayport, Developmental Neuroscience, Columbia University, New York, USA

Tahmina Shirin, Virology, Institute of Epidemiology, Disease Control and Research, Dhaka, Bangladesh

For peer review only

For peer review only - http://bmjopen.bmj.com/site/about/guidelines.xhtml

Motahara Tasneem, Food and Agriculture Organization of the United Nations, Dhaka, Bangladesh

Katie Hampson, Institute of Biodiversity Animal Health & Comparative Medicine, University of Glasgow, Glasgow, United Kingdom

Word Count (excluding title page, abstract, references, figures, tables): 3232

***Correspondence to:**

Ayesha Sania,

Department of Psychiatry, Columbia University Medical Center 1051 Riverside Drive New York, NY, USA

as4823@cumc.columbia.edu

For peer review only

ABSTRACT

Objective: To evaluate the diagnostic performance and feasibility of rapid antigen testing for SARS-CoV-2 detection in low-income communities.

Design: We conducted a cross-sectional community-based diagnostic accuracy study. Community health workers, who were trained and supervised by medical technicians, performed rapid antigen tests on symptomatic individuals, and up to two additional household members, in their households and diagnostic results were calibrated against the gold standard RT-PCR.

Setting: Low-income communities in Dhaka, Bangladesh.

Participants: Between May 19 and July 11, 2021, 1240 nasal and saliva samples were collected from symptomatic individuals and 992 samples from additional household members (up to two from one household).

Results: The sensitivity of rapid antigen tests was 0.68 on nasal samples (95% confidence interval, CI: 0.62-0.73) and 0.41 on saliva (CI: 0.35, 0.46), with specificity also higher on nasal samples (0.98, 95% CI: 0.97-0.99) than saliva (0.87, 95% CI: 0.85, 0.90). Testing up to two additional household members increased sensitivity to 0.71 on nasal samples (CI: 0.65, 0.76), but reduced specificity (0.96, 95% CI: 0.94, 0.97). Sensitivity on saliva rose to 0.48 (95% CI: 0.42-0.54) with two additional household members tested but remained lower than sensitivity on nasal samples. During the study period, testing in these low-income communities increased fourfold through the mobilization of community health workers for sample collection.

Conclusions: Rapid antigen testing on nasal swabs can be effectively performed by community health workers yielding equivalent sensitivity and specificity to the literature. Household testing by community health workers in low-resource settings is an inexpensive approach that can increase testing capacity, accessibility, and the effectiveness of control measures through immediately actionable results.

Strengths and limitations of this study

- This study evaluates the performance of rapid antigen testing at the household level with all specimens collected by community health workers.
- Sensitivity and specificity are reported for the index case and for testing of additional household members.
- Lack of data on Ct values precluded evaluation of the performance of rapid antigen tests according to viral load.
- Information related to symptom duration was not available.

For peer review only - http://bmjopen.bmj.com/site/about/guidelines.xhtml

- Diagnostic performance will depend not only on the proficiency of community health workers collecting specimens, but also the predominant SARS-CoV-2 variant(s) circulating and the incidence in the community.

For peer review only

For peer review only - http://bmjopen.bmj.com/site/about/guidelines.xhtml

INTRODUCTION

Since the start of the coronavirus (COVID-19) pandemic, countries have sought to develop rapid and accessible approaches to diagnostic testing. The capacity for RT-PCR, the gold standard diagnostic for SARS-CoV-2, has ramped up around the world. Nonetheless, limitations remain in many low-resource settings because of the high cost, relatively slow turnaround time, and need for specialized laboratory setup and highly skilled personnel to perform RT-PCR.[1–4] Rapid antigen tests are a promising alternative, as they are inexpensive, fast to perform (typically returning results within 15 minutes), and require only minimal technical skills and infrastructure.[5] Rapid antigen tests can therefore be used for symptomatic testing and as a screening tool to monitor the epidemiological situation. However, research is needed to evaluate the performance of rapid antigen tests and to develop an effective model for their deployment in many low- and middle-income country (LMIC) settings where adoption has been slow.

The diagnostic accuracy of rapid antigen tests has been evaluated in multiple clinical and laboratory studies, reporting high specificity and moderate to high sensitivity.[6-9] A recent meta-analysis of 133 clinical studies reported sensitivity of 76.3% (95% CI: 73.1% to 79.2%), rising to 95.8% (95% CI: 92.3% to 97.8%) when analyses were restricted to samples with high viral load (i.e., Ct values < 25).[10] When the goal of testing is to mitigate transmission, the ideal test would be one that not just identifies the presence of SARS-CoV-2, but identifies those individuals that contribute to virus transmission.[11] Therefore, despite their lower sensitivity compared to the gold standard, and because of the immediacy of their results, as well as their low cost and ease of administration, rapid antigen tests are an ideal method for use in the community. Importantly, the direct administration of rapid antigen tests at households has the potential to lower testing costs and reduce other logistics barriers and risks, including the movement of infectious individuals when seeking tests. However, questions remain around the most appropriate sample type and delivery models for feasible and effective rapid antigen testing in communities.

Many countries that are constrained by limited diagnostic and healthcare infrastructure and human resources have used community health workers to aid service delivery during the pandemic.[12,13] In June 2020, responding to the Bangladesh Preparedness and Response Plan call to emergency action, the Food and Agriculture Organization of the United Nations, the United Nations Populations Fund, and a collective of partner international agencies, non-governmental and civil society organizations (see acknowledgments) initiated a Community Support Team intervention under the leadership of the Bangladesh Government's Directorate General of Health Services (DGHS). Community Support Teams comprised volunteers trained to search and screen symptomatic COVID-19 cases for preventing symptomatic transmission and were mobilized throughout the two city corporations of Dhaka, with an emphasis on socioeconomically deprived areas. Using this network, we evaluated the diagnostic performance and feasibility of rapid antigen testing for the identification of COVID-19 cases in the

For peer review only - http://bmjopen.bmj.com/site/about/guidelines.xhtml

community. Here we report on the sensitivity and specificity of rapid antigen testing by community workers on nasal and saliva samples from symptomatic individuals and their household members, including the impact of testing multiple household members on diagnostic performance. Based on these experiences in Dhaka, we further discuss the potential for community health workers to scale up rapid antigen testing for SARS-CoV-2 in low-income communities.

For peer review only

METHODS

Data collection

Community Support Teams comprised local volunteers that were trained by medical technologists to search for and identify suspected COVID-19 cases through hotline calls and community-based channels, and to counsel them in preventative measures, focusing on mask-wearing, isolation for suspected cases, and quarantine of contacts for 14 days, as well as prescribing over-the-counter medications, and referring cases with severe symptoms to dedicated telemedicine services. At the time of the study, 750 volunteers, hereafter referred to as community health workers, within 465 Community Support Teams were operating across underrepresented vulnerable communities in Dhaka.

Between May 19 and July 11, 2021, study participants (n=1240) were recruited from low-income communities in Dhaka by a subset of community health workers (n=60, split into 30 teams of two persons) who had been trained in sample collection and testing by medical technologists. This subset of community health workers were selected by their line managers based on their performance within the Community Support Teams, where they carry out syndromic surveillance to identify suspected COVID-19 cases and refer them for support. They primarily operated in 15 wards within both the Dhaka North and Dhaka South City Corporations, but sometimes traveled to neighboring wards when potential COVID-19 cases were identified. At that time community health workers were primarily identifying cases through systematic searches within their communities. Community members aged 16 years or older with no previous medical history of bleeding disorders were considered eligible for the study.

Once the study began, index participants were considered eligible for recruitment if, upon screening by the community health worker (guided by a mobile phone application), they were suspected to have COVID-19 infection because they reported with a fever (>38℃ or >37.5℃ following use of antipyretics) or a history of fever and had one or more of the following symptoms: difficulty breathing, cough, diarrhea, headache, loss of smell, loss of taste, muscle pain, red eyes, runny nose, sore throat, tiredness, vomiting, or a wet cough. Participants were then recruited if they consented to be tested. In each household, these suspected COVID-19 cases (index participants) were tested by the community health workers using rapid antigen tests, and then up to two additional household members, regardless of symptoms, were tested. These additional household members were recruited if they met the eligibility criteria (aged 16 or older and no history of bleeding disorder) and were available in the household at the time of testing, with those who had most contact with the index cases prioritized for selection. Community health workers uploaded participants' metadata, including their age, gender, and symptoms as well as rapid antigen test results (see below), to a centralized database via the mobile application. Methods are described in further detail by Chadwick et al.[14]

For peer review only - http://bmjopen.bmj.com/site/about/guidelines.xhtml

From each participant, two mid-turbinate nasal swabs from both nostrils, as well as two oropharyngeal (OP) swabs, and raw saliva were collected. One nasal and OP swab was pooled to create one sample for RT-PCR following protocols described by Vogels et al.[15] Combining nasal and OP swabs for RT-PCR was the standard guideline followed at Bangladesh's Institute of Epidemiology, Disease Control, and Research (IEDCR). The swabs were sent to IEDCR for RT-PCR testing via cold storage, with samples for RT-PCR testing returned immediately using dedicated transportation. The nasal and saliva samples were tested independently with rapid antigen tests (SD Biosensor STANDARD™ Q COVID-19 Ag Test BioNote) in the household. Details of the sample collection and testing procedure described in supplement 1.

In the pilot phase of the study, we evaluated the performance of OP, nasal and saliva swabs among symptomatic cases, which showed the performance of OP swabs slightly better than the nasal swabs. However, there were concerns of injury during OP swabs collected by the community health workers, so the rapid antigen tests were only performed on nasal and saliva swabs. The manufacturer reports that the STANDARD Q COVID-19 Ag Nasal Home Test has a sensitivity of 94.94% (75/79) and specificity of 100% (217/217)[16] and that the STANDARD Q COVID-19 Ag Saliva Home Test has a sensitivity of 94.74% (18/19) and a specificity of 100% (73/73).[17] In clinical settings, the nasal test has a lower sensitivity (66.7% to 100%), but high Kappa values (0.92 to 0.852).[18] Similarly, the saliva test has a lower sensitivity (66.1%), but high specificity (99.6%) in clinical settings.[19]

All participants provided written informed consent for the health screening, collection of personal information and samples, and test results to be analyzed in the study. The study protocol was approved by the Institutional Review Board at the IEDCR, Ministry of Health, Bangladesh, IEDCR/IRB/2021/04.

Patient and public involvement

Participants were not involved in setting the research question or the outcome measures, but participants and their family members were intimately involved in the design and implementation of the study. During consultation in the design phase of the study, community members expressed their preference to be tested in their homes in preference to in a community gathering place. The index patient and their family members received their COVID-19 test results from their trusted community health care workers. They were also advised on isolation and quarantine measures, and they were connected with Government's telemedicine services when they needed.

Statistical analysis

We assessed the distribution of patient age and sex by the numbers of participating household members using mean (SD) and counts (percentages) for continuous and categorical variables,

For peer review only - http://bmjopen.bmj.com/site/about/guidelines.xhtml

respectively. We estimated test sensitivity specificity, positive predictive values (PPV), negative predictive values (NPV) and accuracy of rapid antigen tests with their 95% exact binomial confidence intervals for all samples from index cases, according to sample type.[20] We further compared the sensitivity and specificity of the rapid antigen tests on each swab type and pooled, against RT-PCR, and results were further stratified by the numbers of additional household members tested. We compared group differences using ANOVA and chi-square tests as appropriate.

To examine how the use of community health workers could affect testing capacity we examined changes in the modality of sample collection and testing over time at IEDCR, corresponding to the training of community health workers for this study. Finally, we compared the expected number of positive COVID-19 diagnoses if RT-PCR testing was replaced with rapid antigen testing, based on our estimates of test sensitivity. For this hypothetical comparison we assume a constrained diagnostic testing budget and a cost of $5 per rapid antigen test versus $30 per RT-PCR laboratory diagnosis and we explored a range of prevalence levels within the community.

All analyses were undertaken in R Statistical Software (v4.0.02)[21] and EpiR R package (v2.0.39).[22] All data for the results presented in this paper is available in a repository accessible to the public.[23]

For peer review only - http://bmjopen.bmj.com/site/about/guidelines.xhtml

RESULTS

During the study period, 1240 subjects consented to participate in the study. Of these subjects, 17 were excluded from the analyses due to missing information on the clinical definition used to diagnose a suspected case of COVID-19 (N=9, 0.7%), invalid results from rapid antigen tests on saliva samples or nasal swabs (4, 0.3%), or missing or invalid RT-PCR results (N=4, 0.3%). Table 1 shows the sex and age distribution of the participants by index person (N=1223), first additional household member (N=710), and second additional household member (N=283)

Table 1. Demographic characteristics of the study participants

		Index person (N=1223)	Second household member (N=710)	Third household member (N=283)	Total (N=2216)
**Sex***	Female	575 (47.0%)	397 (55.9%)	151 (53.4%)	1123 (50.7%)
	Male	640 (52.3%)	312 (43.9%)	132 (46.6%)	1084 (48.9%)
**Age***	Mean (SD)	36.522 (14.2)	36.784 (14.42)	34.735 (15.4)	36.377 (14.48)

* Totals may not add up because of missing values.

Rapid antigen tests performed better on nasal than saliva samples

The sensitivity, specificity, positive predictive value (PPV), and negative predictive value (NPV) of rapid antigen tests on nasal swabs and saliva samples are shown in Table 2 and Figure 1 (red bars). Of all subjects with complete test results (N=1223), 291 were RT-PCR positive on pooled OP and nasal samples. Of these, 198 were rapid antigen test positive by nasal swab and 118 were rapid antigen test positive by saliva only sample. Therefore, the overall sensitivity of rapid antigen testing on nasal samples was 0.68 (95% confidence interval, CI: 0.62-0.73) and on saliva samples was 0.41 (95% CI: 0.35-0.46). Saliva samples picked up some RT-PCR-positive subjects who were not positive in the nasal swab (n = 22), and vice versa (n = 102). Specificity of nasal swabs (0.98, 95% CI: 0.97-0.99) was also higher than saliva samples (0.87, 95% CI: 0.85-0.90). The overall accuracy of rapid antigen tests on nasal swabs was higher (0.91 vs 0.76) compared to accuracy on saliva samples (table 2).

Table 2. Performance of rapid antigen tests compared to RT-PCR, among all index household members tested

The total sample size was 1223 index subjects, 95% confidence intervals are shown in brackets. RAT = rapid antigen test, PPV = positive predictive value, NPV = negative predictive value.

	**Nasal sample**<br>PCR + PCR -Total<br>RAT + 198 21 219<br>RAT - 93 911 1004<br>Total 291 932 1223	**Saliva sample**<br>PCR + PCR -Total<br>RAT + 118 117 235<br>RAT - 173 815 988<br>Total 291 932 1223
Sensitivity	0.68 (0.62,0.73)	0.41 (0.35, 0.46)
Specificity	0.98 (0.9, 0.99)	0.87 (0.85, 0.90)
PPV	0.90 (0.86, 0.94)	0.50 (0.44, 0.57)
NPV	0.91 (0.89, 0.92)	0.82 (0.80, 0.85)
Accuracy	0.91 (0.89, 0.92)	0.76 (0.74, 0.79)

For peer review

The probability of a false negative rapid antigen test (i.e., discordant with a positive RT-PCR test) increased with the number of days between self-reported symptom onset and testing, and very few false negatives occurred within the first three days of symptoms (Figure 2). However, our sample size (55 false negative rapid antigen tests from 736 test results with self-reported symptoms onset date) was too small for rigorous quantitative analysis of this relationship.

Testing additional household members only marginally increases sensitivity but decreases specificity

The sensitivity of rapid antigen tests on nasal swabs increased from 0.68 (95% CI: 0.62-0.73) when only the index person was tested, to 0.70 (95% CI: 0.65-0.76) when an additional household member was tested (Figure 1, green bars) and to 0.71 (95% CI: 0.65-0.76) when a second household member was also tested (Figure 1, blue bars), although it should be noted that only a subset of households had additional members available for testing at the time of community health worker visits. With this gain in sensitivity, there was a corresponding drop in

For peer review only - http://bmjopen.bmj.com/site/about/guidelines.xhtml

specificity with additional household members, from 0.98 (95% CI: 0.97-099) to 0.96 (95% CI: 0.94-0.97) for two additional members tested (Figure 1). Similarly, sensitivity of rapid antigen testing on saliva samples increased from 0.42 (95% CI: 0.35-0.46) to 0.48 (95% CI: 0.42-0.54) with two additional household members tested, although there was a corresponding drop in specificity from 0.87 (95%CI: 0.85-0.90) to 0.82 (95% CI: 0.79-0.84). Testing both sample types also yielded a modest increase in sensitivity but a large drop in specificity (Figure 1).

Community health workers achieved higher testing coverage than routine laboratory testing coverage

During the validation phase of this study between May 19, 2021, and July 11, 2021, community health workers collected 73% of the samples from these low-income communities that were tested at IEDCR (Figure 3). Based on the increase in testing volume in these communities and the self-reported experiences of the community health workers involved in this work, we deduced that testing at household level promotes testing. Indeed, the willingness of symptomatic individuals to be tested more than doubled, when testing was offered in people's homes by the community health workers, in comparison to when community health workers offered free testing the next day by medical technologists, which was also in people's homes or at designated local test points.

Shortly after the study began, the Delta variant swept across Bangladesh, and by late June community workers were collecting around 90% of samples in these communities as case numbers surged. The community health workers trained to collect samples for this study continued to collect samples for IEDCR as part of their routine RT-PCR surveillance during this third wave of infection (Figure 3). This period of implementation demonstrated that community health workers can collect and test over 4 times more households compared to the standard approach of community testing (Figure 3), which relies upon follow-up visits by medical technologists the day after symptomatic households are identified by community health workers. Assuming a constrained diagnostic testing budget and a cost of $5 per rapid diagnostic test and of $30 per RT-PCR laboratory diagnosis, we calculate that household (rapid antigen) testing would detect four times more cases than laboratory (RT-PCR) testing as shown in Figure 4, despite its lower sensitivity, under a range of prevalence levels (from <0.05 to >0.4, i.e. spanning the range of test positivity reported from Dhaka in 2021).

For peer review only - http://bmjopen.bmj.com/site/about/guidelines.xhtml

DISCUSSION

We evaluated the performance of rapid antigen testing on nasal and saliva samples collected by community health workers at the household level in low-income communities across Dhaka, Bangladesh. We estimated that rapid antigen tests on nasal swabs had moderate sensitivity and high specificity as expected in a community setting. We also found that testing additional household members increased the sensitivity of detection of COVID-19 infection only marginally, but decreased specificity. This study capitalized on a network of community volunteers who were initially trained to clinically assess individuals reporting COVID-19 symptoms as part of the crisis response and demonstrated that community health workers can administer rapid antigen tests effectively and achieve high coverage in community settings. Our results support recommendations for scaling up rapid testing as part of the COVID-19 response in low-resource settings.

Our report of the superior performance of rapid antigen tests on nasal samples compared to saliva samples confirms the existing literature from clinical and laboratory settings.[24] A recent study reported poor concordance between nasopharyngeal swab specimens and saliva samples (75.9%, kappa coefficient 0.310).[25] However, a meta-analysis examining the performance of RT-PCR concluded that saliva samples were as sensitive as nasopharyngeal swabs.[26,27] A likely explanation for the lower sensitivity of rapid antigen tests on saliva could be due to lower viral load, which would have less impact with RT-PCR.[28] A study on the diagnostic accuracy of rapid antigen tests conducted at testing sites in the Netherlands found that their sensitivity increased from 60% to 85% after applying a viral load cut-off as a proxy for infectiousness.[29] The performance of rapid antigen tests further depends on the virus variant, with a higher viral load reported in saliva for the Beta variant compared to others.[30] Although saliva samples have advantages including easier administration (typically self-collected for testing in many high-income settings), being less invasive, and causing minimal discomfort,[31] we confirm the superiority of nasal swabs in terms of sensitivity and specificity, at least for the Delta variant, which was most prevalent in Dhaka during our study.[32]

Our estimate of rapid antigen test sensitivity on nasal samples (68%) is comparable to that reported by Cerruti et al. (70.6 %) from a mix of symptomatic and asymptomatic participants.[33] Estimates of sensitivity reported by the manufacturer and by studies conducted among symptomatic patients at the point of care are, however, much higher than in our report. SD biosensor, the manufacturer of the STANDARD Q COVID-19 Ag Nasal Home Test reports a sensitivity of 94.94% [16], and an SD biosensor rapid antigen test had a sensitivity of 89.0% at a point-of-care community-based testing center, where the vast majority of the patients were symptomatic (97.8%).[34] Higher sensitivity in laboratory and clinical settings is likely due to higher viral loads. Several prior reports including a recent meta-analysis confirmed that rapid antigen tests have their highest sensitivity in samples with high viral load (Ct value <25)[10] and therefore lower sensitivity is to be expected when testing in the community includes both

For peer review only - http://bmjopen.bmj.com/site/about/guidelines.xhtml

individuals who are currently infectious as well as those who are recovering (Figure 2).[11] Moreover, a point-of-care rapid antigen test study found that when rapid antigen tests and RT-PCR results were discordant, SARS-CoV-2 could not be cultured from the samples, suggesting that these patients were unlikely to be infectious.[35] Thus, from a public health perspective, where curtailing disease transmission is the goal, only people with high viral loads need to be detected as they are capable of transmitting the disease to others.[4,36] Rapid antigen tests are better suited to screening for people with high viral loads, the population which poses the greatest risk for the community. This combined with their reduced costs, logistics, and speed of turnaround makes them advantageous for supporting the rapid and effective implementation of targeted prevention measures.

To the best of our knowledge, this is the first study to report on the performance of rapid antigen testing at the household level. The hypothesis that testing of additional household members could improve sensitivity was motivated by the previous report of higher sensitivity achieved in flock-based testing in animals.[37] For highly pathogenic avian influenza H5N1 in backyard poultry flocks, Robyn et al.[38] demonstrated that combining a standard case definition with one or more rapid antigen tests can increase the sensitivity of case detection to closer to that of the gold standard RT-PCR while maintaining specificity. Depending on the prevalence of COVID-19 cases in the community, testing additional household members may slightly improve sensitivity. However, this strategy will incur higher costs as well as a greater workload for the community health workers. Overall, our findings underscore the importance of testing symptomatic patients in the community.

A limitation of our study is the lack of data on Ct values which precluded evaluation of the performance of rapid antigen tests according to viral load (with Ct values as a proxy). The higher sensitivity and specificity in samples with high viral load have been confirmed in several prior studies.[4,11]. Recent reports indicate that a large proportion of asymptomatic COVID-19 cases also have high viral load [39,40]. Since the eligibility criteria to be an index participant in our study included presence of fever and other covid-like symptoms, the estimates of sensitivity and specificity from our study are not generalizable to asymptomatic cases. Another limitation is that while we found that the probability of false negative results increases as the duration of symptoms increases, our sample size (55 false negative rapid antigen tests from 736 test results with self-reported symptoms onset date) was too small for rigorous quantitative analysis of this relationship. Finally, our study was conducted during a period of high covid transmission in the community and the index participants were symptomatic, indicating a high pretest probability, which impacts the positive and negative predictive values of a test. Therefore, the PPV and NPV in other settings will differ depending on the pretest probability, and the accuracy of rapid tests will also vary with the quality of the training provided to community health workers and their supervision.

For peer review only

For peer review only - http://bmjopen.bmj.com/site/about/guidelines.xhtml

Our study was conducted in a low-resource community setting among a large sample of subjects (n=1240) with all specimens collected by community health workers. These communities are underserved by routine testing procedures in Bangladesh, which require that individuals visit a testing facility and pay for a test (USD15-50 for a RT-PCR with results returned 2-5 days later and more recently USD 5-10 for a rapid antigen test). During the peak of the pandemic, to access care for complications due to COVID-19 patients were required to present a positive test result, and those requiring non-COVID-19 care were required to present with a negative test, affecting the care that individuals could access. Community health workers helped to improve a critical gap in access to testing, which increased the number of people tested from these communities. Moreover, people in these communities were keen to be tested as results were returned immediately in the privacy of their own household. Additionally, familiarity and trust of the community health workers likely contributed to people's willingness to be tested. During the rapid antigen testing validation, the Delta variant began to surge in Dhaka, and community health workers trained to collect samples as part of this study were deployed to continue sample collection for RT-PCR testing. In August 2021 Community Support Teams supported by FAO and UNFPA collected over 80% of the samples from these communities that were tested at the national laboratory, IEDCR, during Bangladesh's third wave, which was the largest peak experienced so far. Sample collection by community health workers proved more efficient and acceptable in these communities and meant that IEDCR was better able to meet testing demand at this critical time.

During the COVID-19 pandemic, many LMIC governments have established community health worker programs to support diagnostic testing, treatment, immunization, and contact tracing.[41] Global initiatives have sought to reduce the costs of rapid antigen tests for COVID-19 to below US$5 each, with catalytic funding to guarantee prices for LMICs.[12, 27] Our study demonstrates that community health workers are capable of effective household-level rapid antigen testing in low-income communities in Dhaka. This provides an example that can be replicated in many community-based programs in Bangladesh and other low resource settings. Future research should evaluate the scalability of rapid testing deployed by community health workers to mitigate COVID-19 in LMIC communities.

Acknowledgments: The authors thank the Directorate General of Health Services (DGHS), Bangladesh, and the Institute of Epidemiology Disease Control and Research (IEDCR) for ongoing SAR-CoV-2 surveillance and prevention measures. The Community Support Team intervention is funded by the World Bank, USAID, and FCDO (UKAID). This multi-sectoral, collaborative initiative is led by the DGHS with technical and operational support from several UN Organizations (FAO, UNFPA, UNICEF, WFP), with community health workers and

For peer review only - http://bmjopen.bmj.com/site/about/guidelines.xhtml

volunteers provided by NGOs (BRAC) and civil society organizations (Youth Platform, Utshargo Foundation, Himu Paribahan, CDP, Platform and Young Bangla). Additional technical support was provided by national institutions such as a2i and icddr,b. We are extremely grateful to the community support team workers who have volunteered throughout this emergency, dedicating time and energy to provide support and response in these communities and without whom this study would not have been possible, as well as to the Zonal Executive Officers, Ward Councilors, Chief Health Officers, Deputy Chief Health Officers and other administrative members of Dhaka North and Dhaka South City Corporations. The University of Glasgow LMICs COVID-19 working group provided valuable feedback and discussion.

Contributors: EB, AS, and KH conceptualized and designed the study, and EB secured funding. AS, EB, TS, ASMA, MR, ANA, FK, and KH planned the study and provided scientific, administrative, and technical support during implementation and data collection of the study. TC developed the standard operating procedures for collection of the samples from the community. MEK developed the data collection tools and managed the data. JA coordinated activities, contributed in data analysis, interpretation of results and drafting of the manuscript. MT led the field implementation, and with ZH trained and supervised the community health workers and provided technical support during data collection. AS wrote the data analysis plan, and the first manuscript draft. SL conducted data analysis, contributed in interpretation of results and revised the manuscript. YKR assisted in literature reviews and manuscript drafting. KH, FC, and DH undertook the data analysis, data interpretation and manuscript revisions. EB, KH, and AS had full access to all of the data in the study and took responsibility for the integrity of the data and the accuracy of the data analysis. All authors reviewed and approved the final manuscript.

Funding: The Bill and Melinda Gates Foundation (BMGF) funded work by FAO (INV-022851) and UoG reports funding from Wellcome (095787/Z/11/Z).

Competing interests: None declared.

Data availability statement: Data and code for replication of the study results are available from: https://zenodo.org/badge/latestdoi/432151026. [23]

Ethics approval: This study was approved by the Institutional Review Board at the IEDCR, Ministry of Health, Bangladesh, IEDCR/IRB/2021/04.

For peer review only

REFERENCES

1 Brihn A, Chang J, OYong K, *et al.* Diagnostic Performance of an Antigen Test with RT-PCR for the Detection of SARS-CoV-2 in a Hospital Setting - Los Angeles County, California, June-August 2020. *MMWR Morb Mortal Wkly Rep* 2021;**70**:702–6. doi:10.15585/mmwr.mm7019a3

2 Corman VM, Landt O, Kaiser M, *et al.* Detection of 2019 novel coronavirus (2019-nCoV) by real-time RT-PCR. *Eurosurveillance* 2020;**25**:2000045. doi:10.2807/1560-7917.ES.2020.25.3.2000045

3 Kucirka LM, Lauer SA, Laeyendecker O, *et al.* Variation in False-Negative Rate of Reverse Transcriptase Polymerase Chain Reaction–Based SARS-CoV-2 Tests by Time Since Exposure. *Ann Intern Med* 2020;:M20-1495. doi:10.7326/M20-1495

4 Larremore DB, Wilder B, Lester E, *et al.* Test sensitivity is secondary to frequency and turnaround time for COVID-19 screening. *Sci Adv* 2021;**7**:eabd5393. doi:10.1126/sciadv.abd5393

5 World Health Organization. Antigen-detection in the diagnosis of SARS-CoV-2 infection using rapid immunoassays: Interim guidance. 2020.

6 Krüttgen A, Cornelissen CG, Dreher M, *et al.* Comparison of the SARS-CoV-2 Rapid antigen test to the real star Sars-CoV-2 RT PCR kit. *J Virol Methods* 2021;**288**:114024. doi:10.1016/j.jviromet.2020.114024

7 Hirotsu Y, Maejima M, Shibusawa M, *et al.* Comparison of automated SARS-CoV-2 antigen test for COVID-19 infection with quantitative RT-PCR using 313 nasopharyngeal swabs, including from seven serially followed patients. *Int J Infect Dis IJID Off Publ Int Soc Infect Dis* 2020;**99**:397–402. doi:10.1016/j.ijid.2020.08.029

8 Pilarowski G, Lebel P, Sunshine S, *et al.* Performance Characteristics of a Rapid Severe Acute Respiratory Syndrome Coronavirus 2 Antigen Detection Assay at a Public Plaza Testing Site in San Francisco. *J Infect Dis* 2021;**223**:1139–44. doi:10.1093/infdis/jiaa802

9 Pray IW. Performance of an Antigen-Based Test for Asymptomatic and Symptomatic SARS-CoV-2 Testing at Two University Campuses — Wisconsin, September–October 2020. *MMWR Morb Mortal Wkly Rep* 2021;**69**. doi:10.15585/mmwr.mm695152a3

10 Brümmer LE, Katzenschlager S, Gaeddert M, *et al.* Accuracy of novel antigen rapid diagnostics for SARS-CoV-2: A living systematic review and meta-analysis. *PLOS Med* 2021;**18**:e1003735. doi:10.1371/journal.pmed.1003735

11 Petersen I, Crozier A, Buchan I, *et al.* Recalibrating SARS-CoV-2 Antigen Rapid Lateral Flow Test Relative Sensitivity from Validation Studies to Absolute Sensitivity for Indicating Individuals Shedding Transmissible Virus. *Clin Epidemiol* 2021;**13**:935–40. doi:10.2147/CLEP.S311977

12 David N, Mash R. Community-based screening and testing for Coronavirus in Cape Town, South Africa: Short report. *Afr J Prim Health Care Fam Med* 2020;**12**:3.

13 Bezbaruah S, Wallace P, Zakoji M, *et al.* Roles of community health workers in advancing health security and resilient health systems: emerging lessons from the COVID-19 response in the South-East Asia Region. *WHO South-East Asia J Public Health* 2021;**10**:41. doi:10.4103/2224-3151.309872

14 Chadwick F, Clark J, Chowdhury S, *et al.* Combining Rapid Antigen Testing and Syndromic Surveillance Improves Community-Based COVID-19 Detection in Low-to-Middle-Income Countries. In Review 2022. doi:10.21203/rs.3.rs-1181429/v1

For peer review only - http://bmjopen.bmj.com/site/about/guidelines.xhtml

15 Vogels CBF, Brito AF, Wyllie AL, *et al.* Analytical sensitivity and efficiency comparisons of SARS-CoV-2 RT–qPCR primer–probe sets. *Nat Microbiol* 2020;**5**:1299–305. doi:10.1038/s41564-020-0761-6
16 SD BIOSENSOR | PRODUCTS. https://www.sdbiosensor.com/product/main?bcode=11&bscode=COVID-19%20Products (accessed 4 Oct 2021).
17 SD BIOSENSOR | PRODUCTS. https://www.sdbiosensor.com/product/product_view?product_no=236 (accessed 14 Oct 2021).
18 Ristić M, Nikolić N, Čabarkapa V, *et al.* Validation of the STANDARD Q COVID-19 antigen test in Vojvodina, Serbia. *PLOS ONE* 2021;**16**:e0247606. doi:10.1371/journal.pone.0247606
19 Igloi Z, Velzing J, Huisman R, *et al.* Clinical evaluation of the SD Biosensor SARS-CoV-2 saliva antigen rapid test with symptomatic and asymptomatic, non-hospitalized patients. *PloS One* 2021;**16**:e0260894. doi:10.1371/journal.pone.0260894
20 Collett D. *Modelling Binary Data*. Boca Raton Florida: : Chapman and Hall/CRC 2002.
21 R Core Team. *R: A language and environment for statistical computing. R Foundation for Statistical Computing*. Vienna, Austria: 2021. https://www.R-project.org/
22 Mark Stevenson, Sergeant E. *epiR: Tools for the Analysis of Epidemiological Data*. 2021.
[dataset] 23 Hampson K. *boydorr/BGD_CST_rapid_testing: BGD CST Rapid Antigen Testing*. Zenodo 2022. doi:10.5281/zenodo.6416157
24 Nagura-Ikeda M, Imai K, Tabata S, *et al.* Clinical Evaluation of Self-Collected Saliva by Quantitative Reverse Transcription-PCR (RT-qPCR), Direct RT-qPCR, Reverse Transcription-Loop-Mediated Isothermal Amplification, and a Rapid Antigen Test To Diagnose COVID-19. *J Clin Microbiol* 2020;**58**:e01438-20. doi:10.1128/JCM.01438-20
25 Uwamino Y, Nagata M, Aoki W, *et al.* Accuracy of rapid antigen detection test for nasopharyngeal swab specimens and saliva samples in comparison with RT-PCR and viral culture for SARS-CoV-2 detection. *J Infect Chemother* 2021;**27**:1058–62. doi:10.1016/j.jiac.2021.04.010
26 Lee RA, Herigon JC, Benedetti A, *et al.* Performance of Saliva, Oropharyngeal Swabs, and Nasal Swabs for SARS-CoV-2 Molecular Detection: a Systematic Review and Meta-analysis. *J Clin Microbiol* 2021;**59**:e02881-20. doi:10.1128/JCM.02881-20
27 Bastos ML, Perlman-Arrow S, Menzies D, *et al.* The Sensitivity and Costs of Testing for SARS-CoV-2 Infection With Saliva Versus Nasopharyngeal Swabs : A Systematic Review and Meta-analysis. *Ann Intern Med* 2021;**174**:501–10. doi:10.7326/M20-6569
28 Procop GW, Shrestha NK, Vogel S, *et al.* A Direct Comparison of Enhanced Saliva to Nasopharyngeal Swab for the Detection of SARS-CoV-2 in Symptomatic Patients. *J Clin Microbiol* 2020;**58**:e01946-20. doi:10.1128/JCM.01946-20
29 Schuit E, Veldhuijzen IK, Venekamp RP, *et al.* Diagnostic accuracy of rapid antigen tests in asymptomatic and presymptomatic close contacts of individuals with confirmed SARS-CoV-2 infection: cross sectional study. *BMJ* 2021;**374**:n1676. doi:10.1136/bmj.n1676
30 Chu CY, Marais G, Opperman C, *et al.* Improved performance of saliva for the detection of the B.1.351 variant in South Africa. 2021. doi:10.1101/2021.03.29.21254563
31 Hung K-F, Sun Y-C, Chen B-H, *et al.* New COVID-19 saliva-based test: How good is it compared with the current nasopharyngeal or throat swab test? *J Chin Med Assoc JCMA* 2020;**83**:891–4. doi:10.1097/JCMA.0000000000000396
32 Saha S, Tanmoy AM, Tanni AA, *et al.* New waves, new variants, old inequity: a continuing COVID-19 crisis. *BMJ Glob Health* 2021;**6**:e007031. doi:10.1136/bmjgh-2021-007031

For peer review only - http://bmjopen.bmj.com/site/about/guidelines.xhtml

33 Cerutti F, Burdino E, Milia MG, *et al.* Urgent need of rapid tests for SARS CoV-2 antigen detection: Evaluation of the SD-Biosensor antigen test for SARS-CoV-2. *J Clin Virol* 2020;**132**:104654. doi:10.1016/j.jcv.2020.104654
34 Berger A, Nsoga MTN, Perez-Rodriguez FJ, *et al.* Diagnostic accuracy of two commercial SARS-CoV-2 antigen-detecting rapid tests at the point of care in community-based testing centers. *PLOS ONE* 2021;**16**:e0248921. doi:10.1371/journal.pone.0248921
35 Torres I, Poujois S, Albert E, *et al.* Evaluation of a rapid antigen test (Panbio™ COVID-19 Ag rapid test device) for SARS-CoV-2 detection in asymptomatic close contacts of COVID-19 patients. *Clin Microbiol Infect* 2021;**27**:636.e1-636.e4. doi:10.1016/j.cmi.2020.12.022
36 Marks M, Millat-Martinez P, Ouchi D, *et al.* Transmission of COVID-19 in 282 clusters in Catalonia, Spain: a cohort study. *Lancet Infect Dis* 2021;:629–36.
37 Martin SW, Shoukri M, Thorburn MA. Evaluating the health status of herds based on tests applied to individuals. *Prev Vet Med* 1992;**14**:33–43. doi:10.1016/0167-5877(92)90082-Q
38 Robyn M, Priyono WB, Kim LM, *et al.* Diagnostic sensitivity and specificity of a participatory disease surveillance method for highly pathogenic avian influenza in household chicken flocks in Indonesia. *Avian Dis* 2012;**56**:377–80. doi:10.1637/9936-091511-Reg.1
39 McEllistrem MC, Clancy CJ, Buehrle DJ, *et al.* SARS-CoV-2 is associated with high viral loads in asymptomatic and recently symptomatic healthcare workers. *PLOS ONE* 2021;**16**:e0248347. doi:10.1371/journal.pone.0248347
40 Hasanoglu I, Korukluoglu G, Asilturk D, *et al.* Higher viral loads in asymptomatic COVID-19 patients might be the invisible part of the iceberg. *Infection* 2021;**49**:117–26. doi:10.1007/s15010-020-01548-8
41 Bhaumik S, Moola S, Tyagi J, *et al.* Community health workers for pandemic response: a rapid evidence synthesis. *BMJ Glob Health* 2020;**5**:e002769. doi:10.1136/bmjgh-2020-002769

For peer review only - http://bmjopen.bmj.com/site/about/guidelines.xhtml

Figure titles and legends:

Figure 1. Sensitivity (left) and specificity (right) of rapid antigen testing (RAT) compared to RT-PCR, when 1 (index person), 2 or 3 members of the same household are tested

Figure 2. The percentage of false negative rapid antigen test results given a positive RT-PCR increases with days from self-reported symptom onset

Figure 3. IEDCR sample collection and testing in Dhaka from May to September 2021
Routine sample collection by IEDCR (Institute of Epidemiology, Disease Control, and Research) from low-income communities in Dhaka where the Community Support Teams operate. Sample collections by medical technologists are shown in grey (MT). The period of rapid antigen testing is indicated, with samples collected for rapid testing in households shown in red (RAT). The subsequent collection of samples by community health workers for RT-PCR testing during the surge of infection driven by the Delta variant is shown in orange. A marked dip in testing in late July corresponded to a religious festival (Eid Al-Adha).

Figure 4. Comparison of positive cases confirmed by rapid antigen testing versus RT-PCR under a specified diagnostic testing budget
We assume that the per cost test of rapid antigen tests (RDT) is \$5 and of RT-PCR is \$30, and the overall testing budget is \$10,000. Modeled results are shown for prevalence of 40% (test positivity exceeded 60% at the peak of the Beta and Delta waves) and of 5% (test positivity rarely dropped below 5% in 2021). Test results are shown based on the estimated rapid antigen test sensitivity and specificity that we reported for testing index cases.

For peer review only

For peer review only - http://bmjopen.bmj.com/site/about/guidelines.xhtml

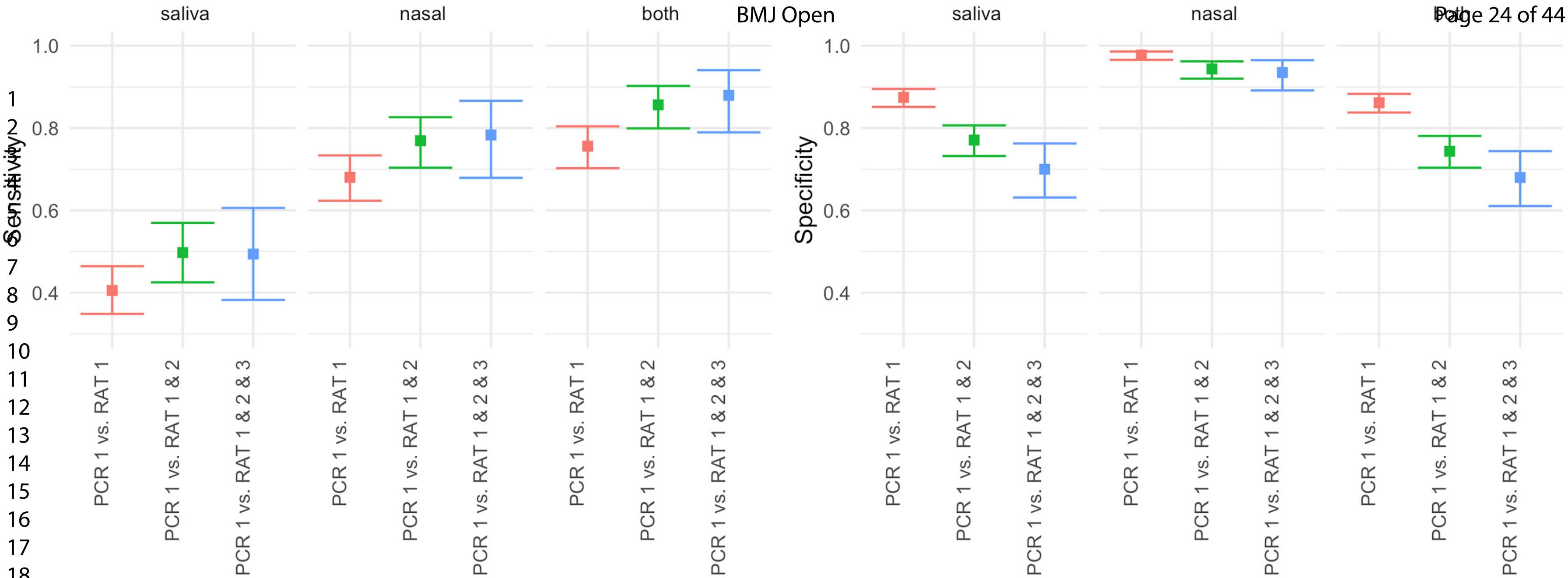
saliva
nasal
both
Sensitivity
1.0
0.8
0.6
0.4
PCR 1 vs. RAT 1
PCR 1 vs. RAT 1 & 2
PCR 1 vs. RAT 1 & 2 & 3
Specificity

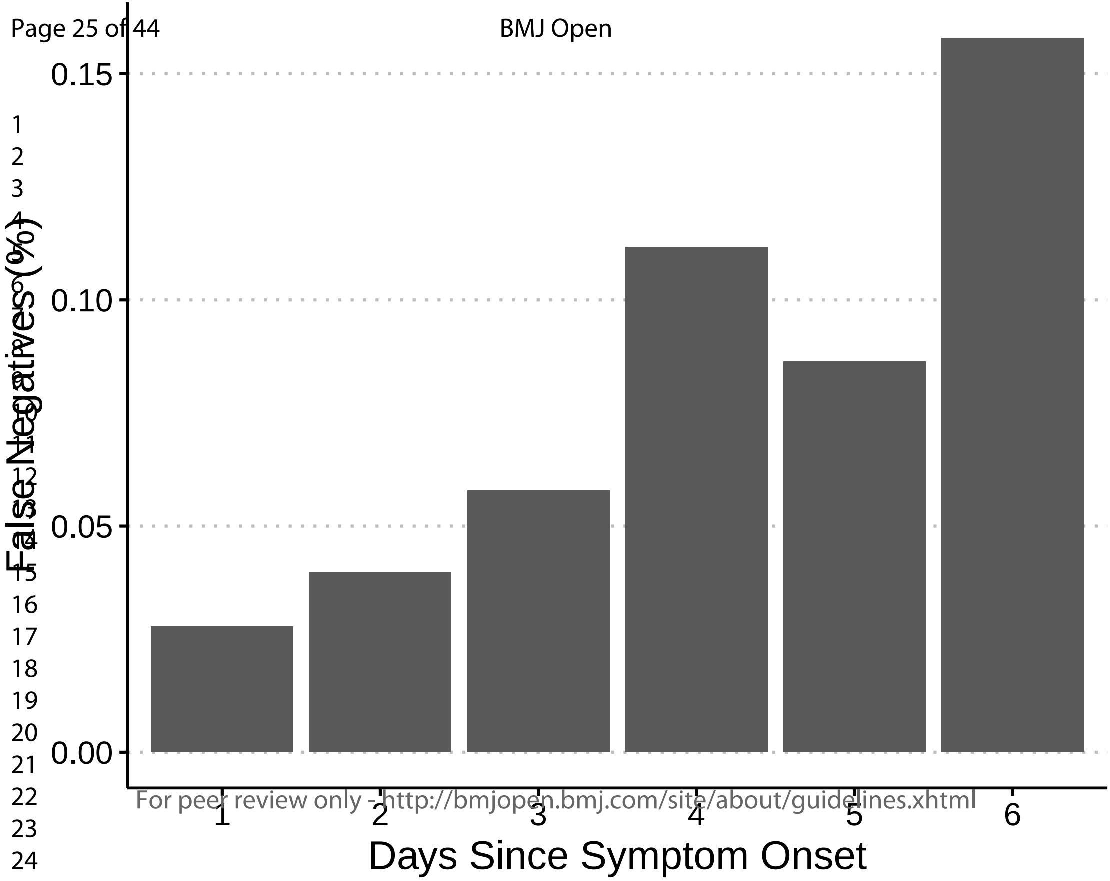
False Negatives (%)
0.15
0.10
0.05
0.00
1
2
3
4
5
6
Days Since Symptom Onset

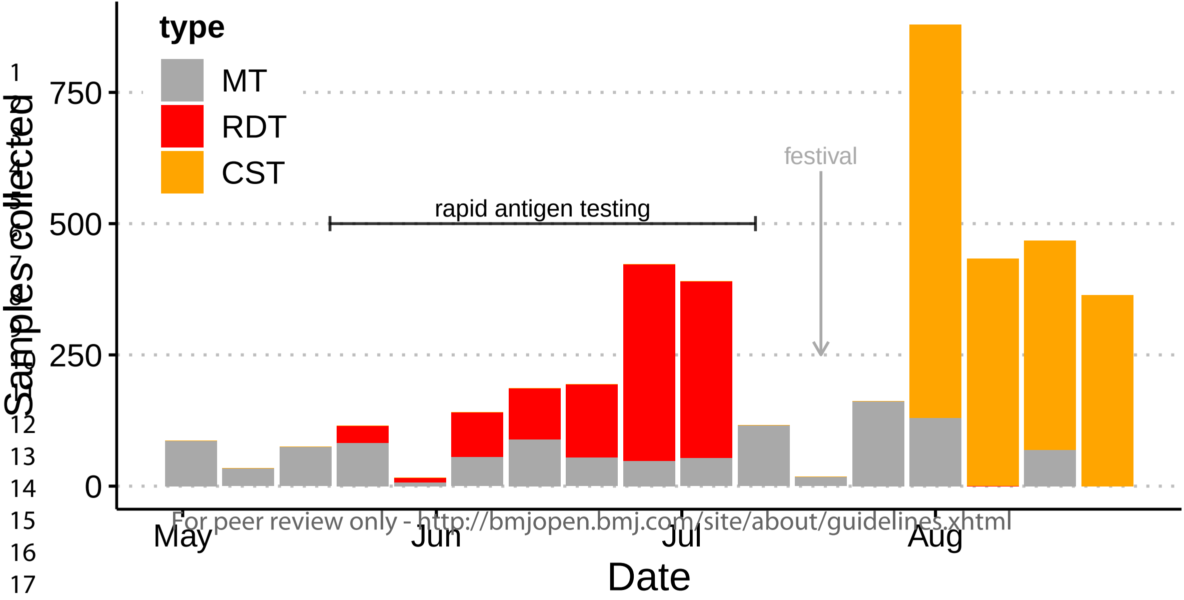

For peer review only - http://bmjopen.bmj.com/site/about/guidelines.xhtml

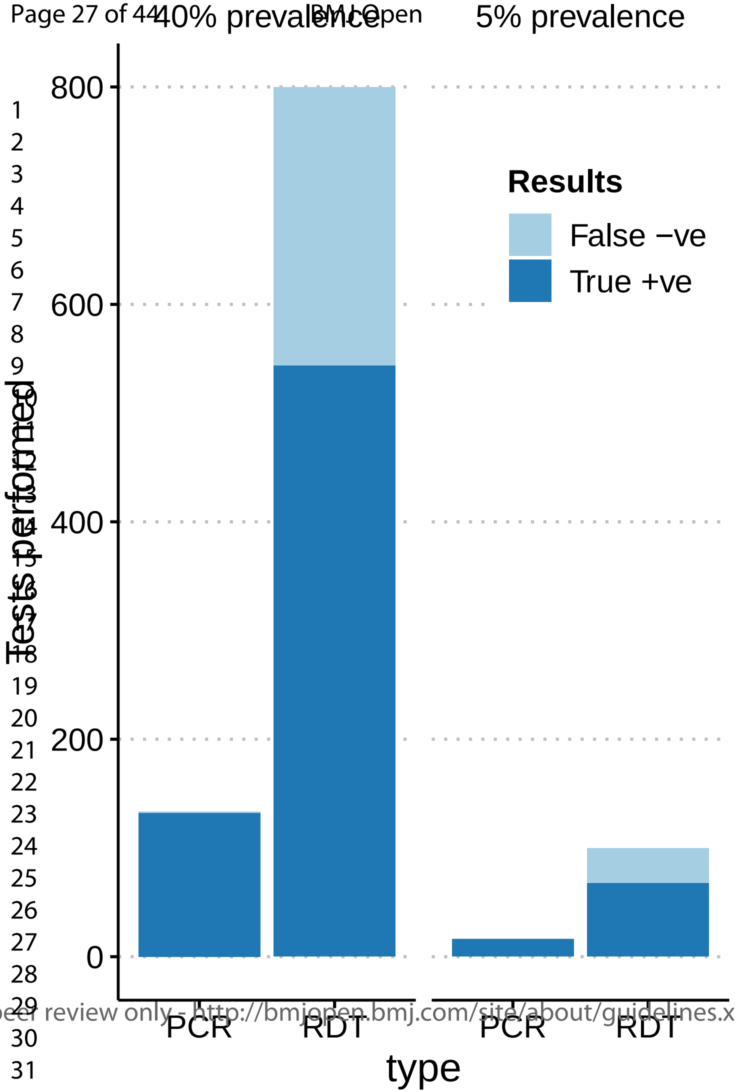
40% prevalence
5% prevalence
Tests performed
800
600
400
200
0
Results
False −ve
True +ve
PCR
RDT
PCR
RDT
type

Overall procedure

1. CSTs will follow their normal routine aiming to find potential virus fighters (PVFs). Once the PVF is found, the CST will perform PVF screening (follow SOP 03) and, if appropriate, VVF screening (follow SOP 05) for verified virus fighter (VVF) identification.
2. If the VVF is found, the team should provide regular support. The VVF and 2 additional household (HH) members (if present) should be informed about the study and the written consent must be obtained before any sample collection.
3. During sample collection and rapid antigen testing the CST team will share responsibilities.
   - CST1 will be collecting rapid test samples (step1) and sample for PCR test (step2) as well as the metadata using the IEDCR app
   - CST2 will perform rapid antigen tests on samples collected by CST1. CST2 will also enter corresponding data into the electronic Kobo forms
4. First, CST1 will collect saliva samples and nasal swabs from all 3 household members (6 samples in total)
5. CST1 will pass the samples (labelled with barcodes and indicating sample type) to CST2 who will perform rapid antigen tests (follow instructions below)
6. While the rapid tests run, CST1 will collect 3 additional samples for PCR (for the VVF and each of the additional HH members) and collect all necessary information (metadata).
7. When the results of rapid test are visible - CST2 will fill out the Kobo form
8. If the rapid test is positive for any of the HH members the household should be instructed to self-isolate . If the rapid test is negative for all HH members the household should also be instructed to quarantine until PCR results are sent (by SMS) and only if the negative result is confirmed can they stop quarantine after 14 days from last exposure .

TIMELINE: after VVF is identified sample collection will follow the workflow:

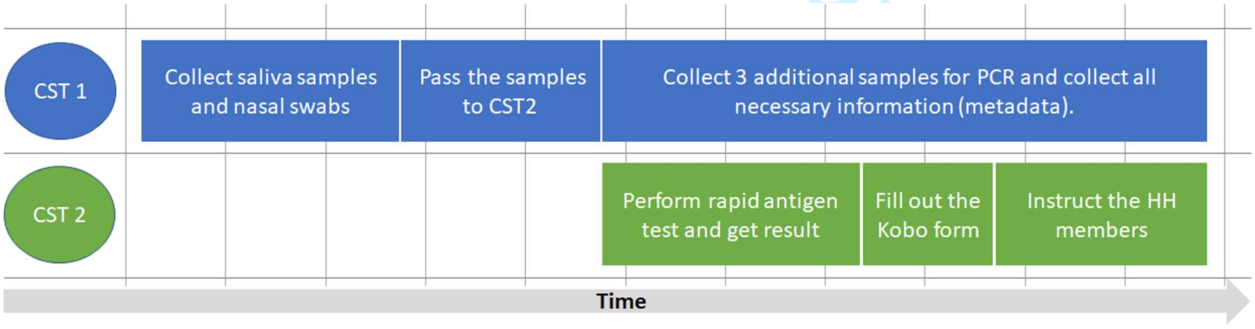

Pre-Requisites

Fifteen (15) MTs will be hired and trained for this phase of the study. Their primary role will be assisting and mentoring CSTs in their activities. After one month of working together the CSTs should gain sufficient confidence to collect samples independently.

For peer review only - http://bmjopen.bmj.com/site/about/guidelines.xhtml

IEDCR's Medical Technologist (MT) will join a CST team (two CST members). CST will follow their daily routine finding PVFs and identifying VVFs by following their SOPs. Once the VVF is identified rapid antigen tests will be performed by the CSTs (on saliva sample and on nasal swab). Additionally, CSTs will collect PCR samples to be tested at the IEDCR and all necessary metadata.

The MT MUST NOT interfere with CST activities. MT should ensure all samples are collected properly. MT should be a mentor for the CSTs, whose aim is to become independent after one month's time period. MT can advise the CST on rapid antigen results interpretation but MUST NOT perform the test.

Considering that the above prerequisites are met, the rest of this document describes the step-by-step procedures for the CSTs and MTs to collect, record, store, transport, and test the samples as well as report the results. In this document, subjects requesting a test will be referred to as ***patients***, while the patients testing positive with RT-PCR for COVID-19 will be referred to as ***cases***.

Preparation

Preparation

Before leaving for sample collection, the MTs/CSTs will collect the following from the IEDCR office.

☐	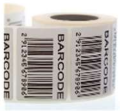	Eight (8) printed copies of seven (7) digit barcode labels for each allocated patient (1 for labeling the Viral transporting medias (VTMs) for PCR testing, 3 for the saliva collection tube, extraction buffer tube (salive to be added into) and the corresponding saliva rapid test, 2 for the nasal swab extraction buffer tube and the corresponding rapid antigen test, 2 for the informed consent forms)
☐	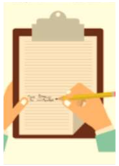	Informed consent forms to get the patient's consent. Put a barcode label on the top right corner of the form.
☐	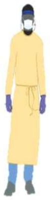	Sealed PPEs (1 for attending each patient)

For peer review only

☐	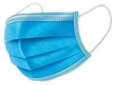	Disposable masks
☐	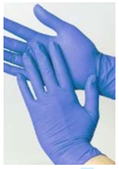	Disposable gloves
☐	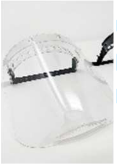	Face shield
☐	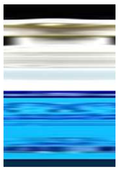	Disinfectant
☐	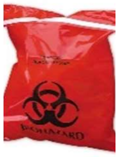	Biohazard bag for disposal of used PPEs, gloves, masks
☐	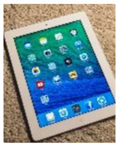	A digital tab for inserting the patients' information into the app
☐	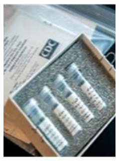	VTMS
☐	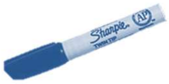	marker for labeling VTMs, tubes and rapid tests

For peer review only

☐	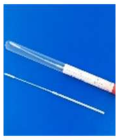	Nasal and throat swab collectionkits
☐	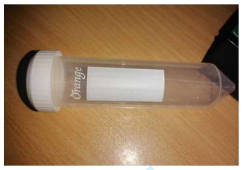	Saliva collection kits (screw cap container)

During the household visit the CST team will follow their routine and perform the following activities:

- PVF screening
- VVF identification

If the PVF (<u>one per HH</u>) is identified as VVF the CST1 will collect their saliva and nasal swab samples and perform rapid antigen tests. In addition, the CST will collect PCR samples (O+N swabs). Rapid antigen and PCR samples will be collected from additional two members of the household who are present during the visit (<u>giving priority to those with any covid-like symptoms and/or to those with being the closest contacts of the VVF</u>).

Explaining the study and collecting the consent

☐	CST will inform that this is a routine test from IEDCR to identify covid-19 cases. In addition to the routine nasal and throat samples (PCR), two more samples will be collected: nasal sample and saliva sample
☐	Notify that the participation is voluntary. If a patient refuses to participate/give additional samples, the routine sample collection procedure will be carried out and all information will be collected in the **IEDCR case selection app** in the digital tab.
☐	Notify that the collection of biospecimens does not pose any potential safety risk.
☐	Notify that these tests do not usually hurt but may cause a little discomfort.
☐	Notify that the maintenance of data confidentiality will be strictly followed, and restrictions on access to data forms will be ensured.

For peer review only

Notify that the patient will be informed about the PCR result by text message or email.

☐ If the rapid test is positive for any of the HH members the household should be instructed to quarantine. If the rapid test is negative, the household should be instructed to self-isolate until PCR results are sent (by SMS) and only if all are negative can they stop isolation.

Labelling sample collection tubes and tests

After obtaining the consent, CST2 will label

- 1,2 or 3 (depending on number of HH members to be tested) rapid antigen tube/s (extraction buffer tube/s) with different barcodes.And also label the tube/s with “+S” (which refers to saliva sample).
- Appropriate number of rapid antigen tubes (extraction buffer tubes) with the corresponding barcodes. And also label the tube/s with “+N” (which refers to nasal sample).
- Same number of falcon tubes (large ones for saliva collection) with the corresponding barcodes
- Same number of VTMs (for PCR sample collection) with the corresponding barcodes
- The sample collection tubes with the VVF’s and HH members’ names to prevent any confusion.
- Using corresponding barcodes rapid antigen tests will be labelled as well (adding S for saliva and N for nasal swab sample)

PPE

CST1 and CST2 will now

☐ **disinfect hands**

CST1 will wear the PPE from the sealed bag, disposable mask, and face shield.

For peer review only

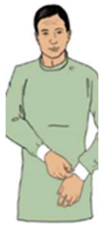

☐ PPE gown

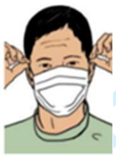

☐ Surgical/N95 mask

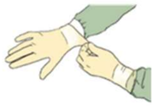

☐ Disposable gloves

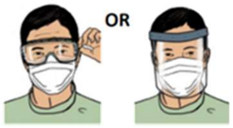

☐ Face shield or goggles

Source: https://www.dph.illinois.gov/sites/default/files/COVID19/COVID19%20sample%20collection%20procedures_5.31.20.pdf
https://www.who.int/csr/disease/plague/collecting-sputum-samples.PDF

For peer review only

Raw Saliva Sample Collection Protocol for Phase 2 (to be collected by CST1)

CST1 should confirm that the patient had no food or drinks before sample collection. Ideally water should be avoided for 10 mins prior to collection and drinks/food/nasal sprays for half an hour before collection. While the patient is getting prepared for the process, CSTs will prepare as well (see above). That way if the patient already had a meal, it will create a time difference for sample collection.

Raw saliva sample collection instruction for CST

The CST2 will hand in the falcon tube with pateind code (RAT 0XXX S) to CST1 for sample collection

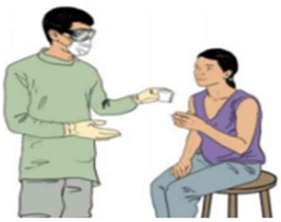

- ☐ Instruct the patient to think about saliva inducing food such as tamarind and begin pooling saliva in their mouth, the patient can gently massage their cheeks to help the process.
- ☐ Request the patient not to cough and produce sputum in the mouth.
- ☐ Labelled (RAT0XXX S) sterile, leak-proof screw cap containers will be handled to the patient.
- ☐ Patient will be instructed to not to touch the inside of the container.

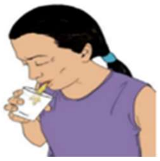

- ☐ Patient will be instructed to gently expel saliva into the container.

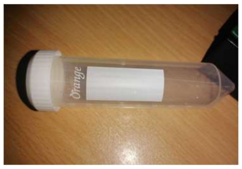

- ☐ Collect 1-5 mL of saliva.

For peer review only

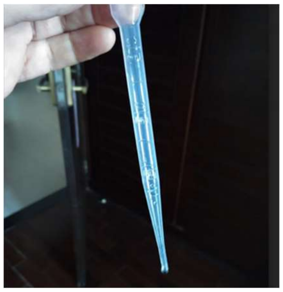

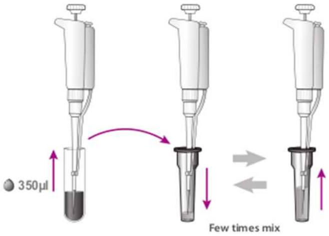

☐ Using an eyedropper collect ~350µl of saliva from the collection tube into the extraction buffer tube (kit) . Mix with an extraction buffer.

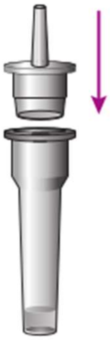

☐ Press the nozzle cap tightly onto the tube

☐ Hand the sample to CST2 to perform the test

For peer review only - http://bmjopen.bmj.com/site/about/guidelines.xhtml

Nasal Swab Sample Collection Protocol for Phase 2 (to be collected by CST1)

Nasal sample collection (infographics showing Left Nasal sample collection) instruction for CST

The CST2 will hand in the extraction buffer tube with patient code (RAT 0XXX N) to CST1 for sample collection

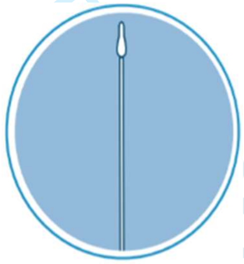

- ☐ Take out the nasal mid-turbinate swab from the packet and keep the tube safely for the time being.
- ☐ Touch only the plastic shaft not the padded end.

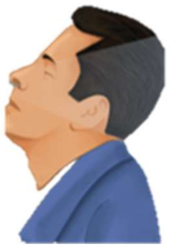

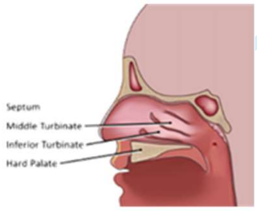

- Ask the patient to sit straight and tilt the head back (approximately 70 degree).

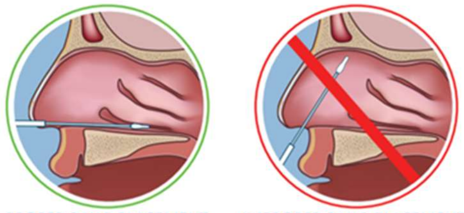

PROPER SWAB PLACEMENT IMPROPER SWAB PLACEMENT

- Insert the swab in the RIGHT nasal spare parallel to the hard palate.
- Resistance will be felt and that is the confirmation of reaching to the nasopharynx.

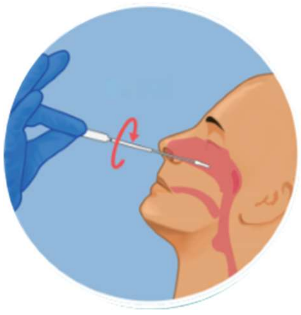

- Once the swab is against the hard surface rotate it several times.

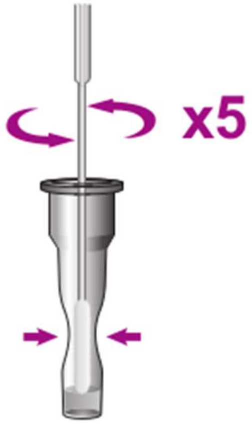

- ☐ Make sure the tube is labelled correctly (RATXXX N, where the barcode corresponds to the right patient)
- ☐ Insert the swab into an extraction buffer tube. While squeezing the buffer tube, stir the swab more than 5 times.
- ☐ Remove the swab while squeezing the sides of the tube to extract the liquid from the swab.

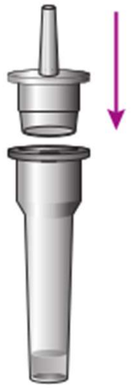

- ☐ Press the nozzle cap tightly onto the tube.
- ☐ Sample to be handed to the CST2

Image and Information Sources:

https://www.cdc.gov/coronavirus/2019-ncov/downloads/lab/NMT_Specimen_Collection_Infographic_FINAL_508.pdf

Rapid antigen test and interpretation of the results (to be performed by CST2)

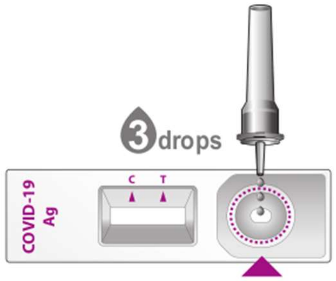

- CST2 will apply 3 drops of extracted specimen to the specimen well of the test device
- double check that the barcode on the tube and n the test is the same (!)

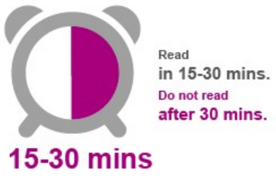

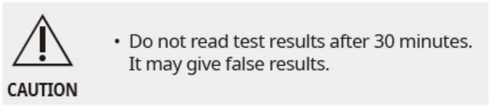

- Read the test result in 15-30 minutes.

Results Interpretation

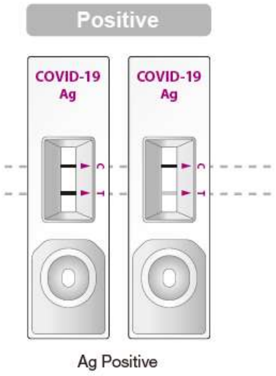

- o Presence of a second colored band, “T” test line, in conjunction with the “C” Control line is always considered as positive. **<u>Even if the “T” test line is faint.</u>**
- o A colored band, control line (C), in the top section of the result window will appear in positive and negative test results.

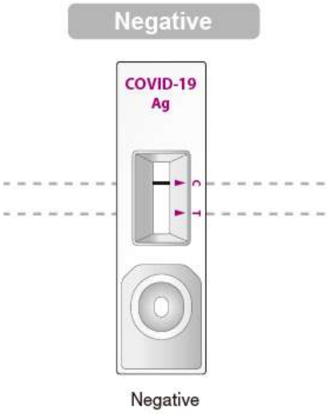

- Presence of only the "C" control line without the "T" test line will be considered as negative.

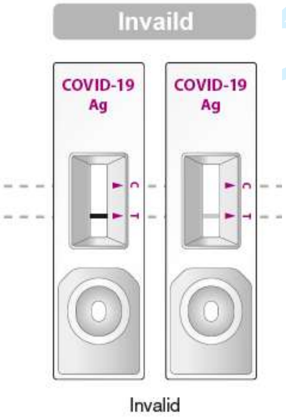

- Absence of the control line in the top section will always consider the result as invalid.

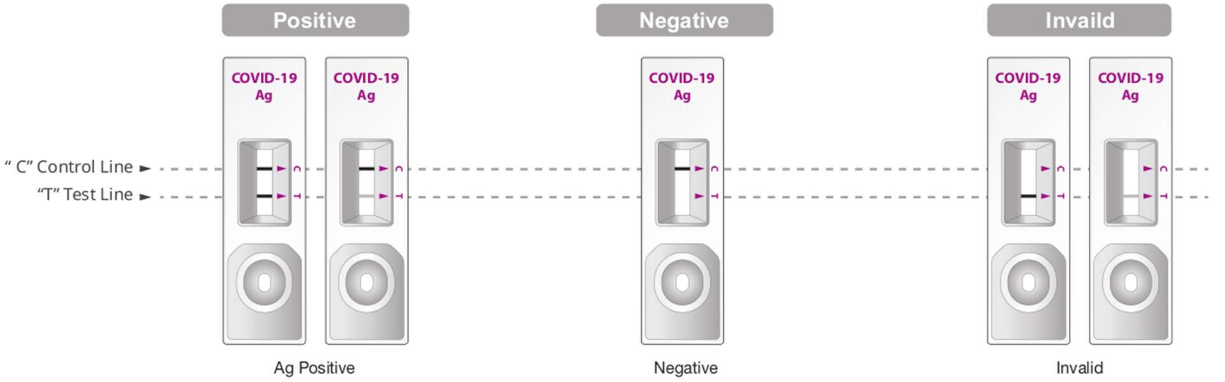

☐ Once both tests are performed and the results are read, CST2 will use Kobo form to register the results, which will involve taking pictures of the tests with visible barcodes for the record.

For peer review only - http://bmjopen.bmj.com/site/about/guidelines.xhtml

PCR Sample Collection Protocol for Phase 2 (to be collected by CST1)

PCR sample contains two swabs (OP/throat and nasal), collected into one VTM tube and transported to the IEDCR for PCR test.

Demographic data

Before sample collection, the CST1 will start collecting demographic information in the digital tablet. It is recommended that CST ensure social distance from the patients and keep wearing PPE the entire time during the data collection.

Table: Procedure for inserting information in the digital tablet is as follows:

- ☐ Open the ***RAT study***, ***IEDCR Case selection*** app in the tab.
- ☐ Start a new form.
- ☐ Enter the barcode.
- ☐ Collect and enter patient-specific information in the form. The form contains fields related to patient demography, list of symptoms, date of onset, and any other relevant information. See the screen shots of the form below.
- ☐ Make sure that all available information is collected from the patient and inserted into the form.

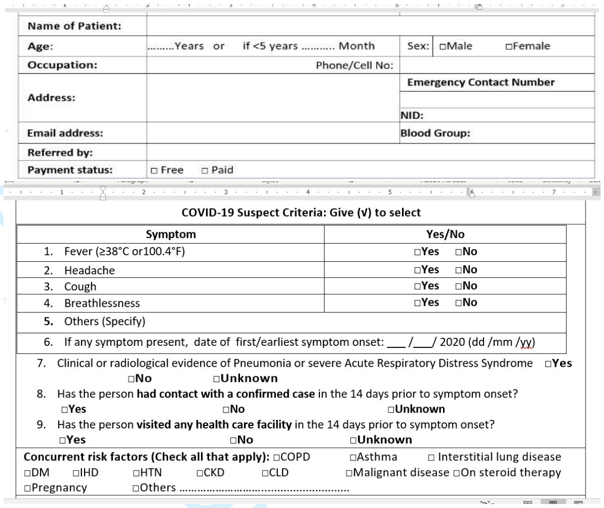

**Name of Patient:**			
**Age:**	……..Years or if <5 years ………. Month	Sex:	□Male □Female
**Occupation:**	Phone/Cell No:		
**Address:**		**Emergency Contact Number**	
		**NID:**	
**Email address:**		**Blood Group:**	
**Referred by:**			
**Payment status:**	□ Free □ Paid		

COVID-19 Suspect Criteria: Give (√) to select

**Symptom**	**Yes/No**
1. Fever (≥38°C or100.4°F)	□**Yes** □**No**
2. Headache	□**Yes** □**No**
3. Cough	□**Yes** □**No**
4. Breathlessness	□**Yes** □**No**
**5.** Others (Specify)	
6. If any symptom present, date of first/earliest symptom onset: ___ /___/ 2020 (dd /mm /yy)	

7. Clinical or radiological evidence of Pneumonia or severe Acute Respiratory Distress Syndrome □**Yes** □**No** □**Unknown**
8. Has the person **had contact with a confirmed case** in the 14 days prior to symptom onset? □**Yes** □**No** □**Unknown**
9. Has the person **visited any health care facility** in the 14 days prior to symptom onset? □**Yes** □**No** □**Unknown**

Concurrent risk factors (Check all that apply): □COPD □Asthma □ Interstitial lung disease □DM □IHD □HTN □CKD □CLD □Malignant disease □On steroid therapy □Pregnancy □Others ………………………………………………

STEP1: OP / Throat swab collection instruction for CST1 (see the infographic below)

The CST2 will hand in the VTM with pateind code (RAT 0XXX) to CST1 for sample collection

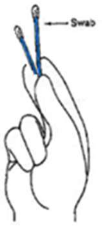

- ☐ Take out one swab from the packet and keep the tubes safely for the time being.
- ☐ Touch only the plastic shafts not the padded ends.

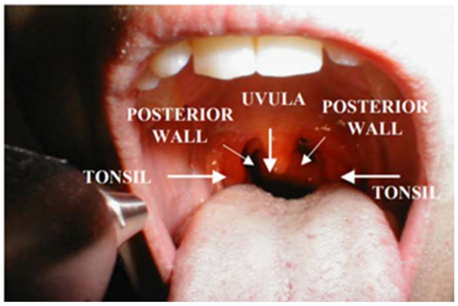

- ☐ Ask the patient to sit straight and tilt the head back.
- ☐ Instruct the patient to widely open the mouth and say “Ahh…”
- ☐ If needed use a sterile tongue depressor to get a better view of the throat.

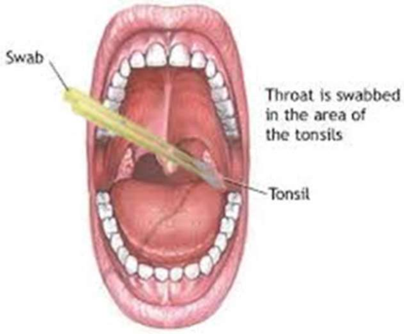

- ☐ Without touching the teeth, gum, tongue, or palate, insert the swabs together to the tonsil and posterior pharynx of any one side.
- ☐ Rub the swabs in the tonsillar area and posterior pharynx, rotate it for 2 times.

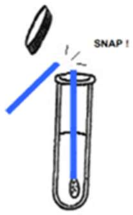

- ☐ After collecting the sample insert the swab into a separate VTMs, label with patient’s barcode
- ☐ Make sure the liquid transport medium covers the tip of the swabs.
- ☐ Break the swab shafts at the marking on the shaft.

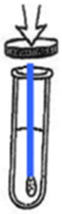

- ☐ Screw the cap back on the test tube tightly.
- ☐ Keep the tube, waiting for inserting a nasal sample for the RT-PCR testing.

STEP2: Nasal sample collection (infographics showing Left Nasal sample collection) instruction for CST1

For peer review only

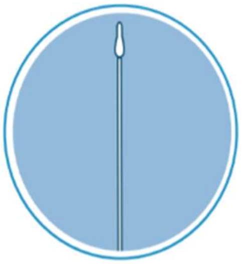

☐ Take out the nasal mid-turbinate swab from the packet and keep the tube safely for the time being.

☐ Touch only the plastic shaft not the padded end.

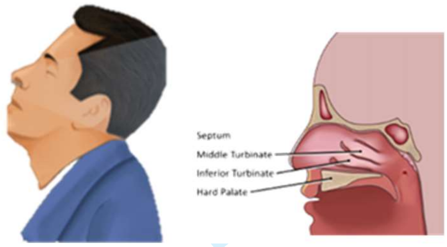

☐ Ask the patient to sit straight and tilt the head back (approximately 70 degree).

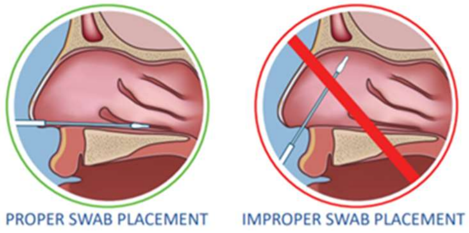

☐ Insert the swab in the LEFT nasal spare parallel to the hard palate.

☐ Resistance will be felt and that is the confirmation of reaching to the nasopharynx.

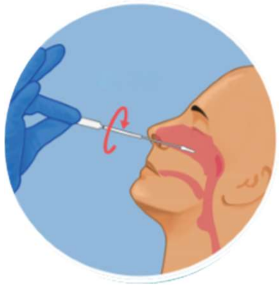

☐ Once the swab is against the hard surface rotate it several times.

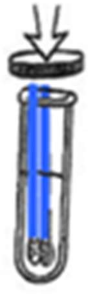

☐ Take out the swab from the left nose and insert the swab into the VTM labelled as "T+N" which already contains the throat swab.

☐ Make sure the liquid transport medium covers the tip of the swabs.

☐ Break the swab shafts at the marking on the shaft.

☐ Screw the cap back on the test tube tightly.

☐ Keep the test tube in the storage box. This combined sample will go for RT-PCR testing

☐ Once the sample is collected, CST1 will check the box in the app (See example below).

Specimen:

☐ Collected ☐ Not collected

If collected mention type:

☐ Combined left nasal swab and throat swab ☐ Nasal swab ☐ Saliva

Image and Information Sources: https://www.cdc.gov/coronavirus/2019-ncov/downloads/lab/NMT_Specimen_Collection_Infographic_FINAL_508.pdf

Sample Store and Transport

- All test tubes/vials to be stored in a Cold Box at 2-8C.
- The samples to be returned to the IEDCR lab at the end of the day.
- Upon return, the storage box will be handled by the lab technicians who run the PCR tests.

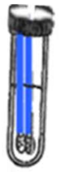

☐ Only the VTM with double swabs, labelled as "T+N",will go for the RT-PCR testing.

For peer review only

At the end of sample collection

CSTs and MT should do the following:

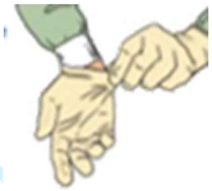	☐ Discard the gloves.
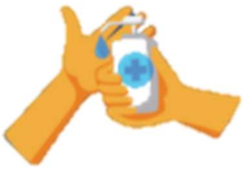	☐ Disinfect the hands with the sanitizer.
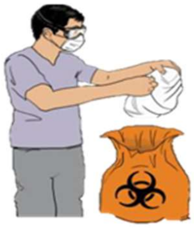	☐ Before entering the transport, CST and MT will discard disposable masks, gloves, PPE in the biodegradable bag. Also disinfect hands as well as the face shield which will be used for the next visit.
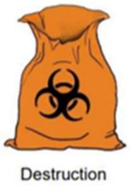	☐ CST and MT must remember that the biodegradable bag needs to be destroyed in the designated place at the IEDCR office.

Image and Information Sources: https://www.who.int/csr/disease/plague/collecting-sputum-samples.PDF

For peer review only - http://bmjopen.bmj.com/site/about/guidelines.xhtml

STROBE Statement—checklist of items that should be included in reports of observational studies

	Item No	Recommendation	Page No
**Title and abstract**	1	(*a*) Indicate the study's design with a commonly used term in the title or the abstract	4
		(*b*) Provide in the abstract an informative and balanced summary of what was done and what was found	4
**Introduction**			
Background/rationale	2	Explain the scientific background and rationale for the investigation being reported	5
Objectives	3	State specific objectives, including any prespecified hypotheses	6
**Methods**			
Study design	4	Present key elements of study design early in the paper	7
Setting	5	Describe the setting, locations, and relevant dates, including periods of recruitment, exposure, follow-up, and data collection	7
Participants	6	(*a*) *Cohort study*—Give the eligibility criteria, and the sources and methods of selection of participants. Describe methods of follow-up<br>*Case-control study*—Give the eligibility criteria, and the sources and methods of case ascertainment and control selection. Give the rationale for the choice of cases and controls<br>*Cross-sectional study*—Give the eligibility criteria, and the sources and methods of selection of participants	7
		(*b*) *Cohort study*—For matched studies, give matching criteria and number of exposed and unexposed<br>*Case-control study*—For matched studies, give matching criteria and the number of controls per case	
Variables	7	Clearly define all outcomes, exposures, predictors, potential confounders, and effect modifiers. Give diagnostic criteria, if applicable	7, 8
Data sources/ measurement	8*	For each variable of interest, give sources of data and details of methods of assessment (measurement). Describe comparability of assessment methods if there is more than one group	*7,8*
Bias	9	Describe any efforts to address potential sources of bias	13
Study size	10	Explain how the study size was arrived at	9
Quantitative variables	11	Explain how quantitative variables were handled in the analyses. If applicable, describe which groupings were chosen and why	8
Statistical methods	12	(*a*) Describe all statistical methods, including those used to control for confounding	8
		(*b*) Describe any methods used to examine subgroups and interactions	8
		(*c*) Explain how missing data were addressed	
		(*d*) *Cohort study*—If applicable, explain how loss to follow-up was addressed<br>*Case-control study*—If applicable, explain how matching of cases and controls was addressed<br>*Cross-sectional study*—If applicable, describe analytical methods taking account of sampling strategy	8
		(*e*) Describe any sensitivity analyses	

Continued on next page

For peer review only - http://bmjopen.bmj.com/site/about/guidelines.xhtml

For peer review only

**Results**			
Participants	13*	(a) Report numbers of individuals at each stage of study—eg numbers potentially eligible, examined for eligibility, confirmed eligible, included in the study, completing follow-up, and analysed	9
		(b) Give reasons for non-participation at each stage	
		(c) Consider use of a flow diagram	
Descriptive data	14*	(a) Give characteristics of study participants (eg demographic, clinical, social) and information on exposures and potential confounders	9
		(b) Indicate number of participants with missing data for each variable of interest	
		(c) *Cohort study*—Summarise follow-up time (eg, average and total amount)	
Outcome data	15*	*Cohort study*—Report numbers of outcome events or summary measures over time	*9*
		Case-control study—Report numbers in each exposure category, or summary measures of exposure	
		Cross-sectional study—Report numbers of outcome events or summary measures	
Main results	16	(*a*) Give unadjusted estimates and, if applicable, confounder-adjusted estimates and their precision (eg, 95% confidence interval). Make clear which confounders were adjusted for and why they were included	10
		(*b*) Report category boundaries when continuous variables were categorized	
		(*c*) If relevant, consider translating estimates of relative risk into absolute risk for a meaningful time period	
Other analyses	17	Report other analyses done—eg analyses of subgroups and interactions, and sensitivity analyses	
**Discussion**			
Key results	18	Summarise key results with reference to study objectives	12
Limitations	19	Discuss limitations of the study, taking into account sources of potential bias or imprecision. Discuss both direction and magnitude of any potential bias	13
Interpretation	20	Give a cautious overall interpretation of results considering objectives, limitations, multiplicity of analyses, results from similar studies, and other relevant evidence	13
Generalisability	21	Discuss the generalisability (external validity) of the study results	13
**Other information**			
Funding	22	Give the source of funding and the role of the funders for the present study and, if applicable, for the original study on which the present article is based	15

*Give information separately for cases and controls in case-control studies and, if applicable, for exposed and unexposed groups in cohort and cross-sectional studies.

Note: An Explanation and Elaboration article discusses each checklist item and gives methodological background and published examples of transparent reporting. The STROBE checklist is best used in conjunction with this article (freely available on the Web sites of PLoS Medicine at http://www.plosmedicine.org/, Annals of Internal Medicine at http://www.annals.org/, and Epidemiology at http://www.epidem.com/). Information on the STROBE Initiative is available at www.strobe-statement.org.

For peer review only

For peer review only - http://bmjopen.bmj.com/site/about/guidelines.xhtml
